# A PfSPZ vaccine immunization regimen equally protective against homologous and heterologous controlled human malaria infection

**DOI:** 10.1038/s41541-022-00510-z

**Published:** 2022-08-23

**Authors:** Benjamin Mordmüller, Zita Sulyok, Mihály Sulyok, Zsofia Molnar, Albert Lalremruata, Carlos Lamsfus Calle, Patricia Granados Bayon, Meral Esen, Markus Gmeiner, Jana Held, Henri-Lynn Heimann, Tamirat Gebru Woldearegai, Javier Ibáñez, Judith Flügge, Rolf Fendel, Andrea Kreidenweiss, Natasha KC, Tooba Murshedkar, Sumana Chakravarty, Pouria Riyahi, Peter F. Billingsley, L. W. Preston Church, Thomas L. Richie, B. Kim Lee Sim, Stephen L. Hoffman, Peter G. Kremsner

**Affiliations:** 1 Institut für Tropenmedizin, Universitätsklinikum Tübingen, Tübingen, Germany; 2 Deutsches Zentrum für Infektionsforschung, Standort Tübingen, Tübingen, Germany; 3 Centre de Recherches Médicales de Lambaréné, Lambaréné, Gabon; 4 Department of Medical Microbiology, Radboud University Medical Center, Nijmegen, The Netherlands; 5 Sanaria Inc., Rockville, MD USA; 6 Protein Potential, LLC, Rockville, MD USA

**Keywords:** Malaria, Live attenuated vaccines

## Abstract

Immunization with radiation-attenuated *Plasmodium falciparum* (Pf) sporozoites (SPZ) in PfSPZ Vaccine, has provided better vaccine efficacy (VE) against controlled human malaria infection (CHMI) with the same parasites as in the vaccine (homologous) than with genetically distant parasites (heterologous). We sought to identify an immunization regimen that provided similar VE against CHMI with homologous and heterologous Pf for at least 9 weeks in malaria-naïve adults. Such a regimen was identified in part 1 (optimization), an open label study, and confirmed in part 2 (verification), a randomized, double-blind, placebo-controlled study in which VE was assessed by cross-over repeat CHMI with homologous (PfNF54) and heterologous (Pf7G8) PfSPZ at 3 and 9–10 weeks. VE was calculated using Bayesian generalized linear regression. In part 1, vaccination with 9 × 10^5^ PfSPZ on days 1, 8, and 29 protected 5/5 (100%) subjects against homologous CHMI at 3 weeks after the last immunization. In part 2, the same 3-dose regimen protected 5/6 subjects (83%) against heterologous CHMI at both 3 and 9–10 weeks after the last immunization. Overall VE was 78% (95% predictive interval: 57–92%), and against heterologous and homologous was 79% (95% PI: 54–95%) and 77% (95% PI: 50–95%) respectively. PfSPZ Vaccine was safe and well tolerated. A 4-week, 3-dose regimen of PfSPZ Vaccine provided similar VE for 9–10 weeks against homologous and heterologous CHMI. The trial is registered with ClinicalTrials.gov, NCT02704533.

## Introduction

The large international investment in malaria control beginning about 2000, resulted in reduction in numbers of clinical cases and deaths caused by malaria by 19 and 50%, respectively by 2014^1^. However, from 2015 to 2019, the numbers of annual cases (229 million in 2019) and deaths (409,000 in 2019) were not reduced^1^. Our current tools are not sufficient to halt transmission and eliminate malaria in the most affected parts of the world; the World Health Organization reported that in 2020 there were more deaths from malaria than from COVID-19 in sub-Saharan Africa^2^.

Malaria is also a significant travel-related disease with an average of 1469 hospitalizations and 11 deaths per year in the United States (US) between 2000 and 2014 and greater numbers in Europe (8349 cases in 2018)^3,4^, most acquired in Africa^5^. Chemoprophylaxis is available, but uptake and compliance are are often inadequate leading to cases and deaths caused by malaria^3–6^.

Vaccines would be powerful antimalarial tools. However, the parasites that cause malaria are much more complex than the viruses and bacteria against which we have vaccines. There is no marketed vaccine in Europe, the US or Africa for a human disease caused by parasites. The most studied malaria vaccine and the closest to marketing is RTS,S/AS01^7^, a subunit recombinant protein vaccine based on the *Plasmodium falciparum* (Pf) circumsporozoite protein (CSP). The Phase 3 program raised questions about the risk-benefit ratio and to provide further data, RTS,S/AS01 was assessed in a pilot implementation program in 9 month-olds in Africa^8^. The pilot program in more than 500,000 infants showed a significant reduction of 30% in severe malaria cases and a significant reduction of 21% in hospitalizations with malaria but no significant reduction in other malaria parameters. The four-dose regimen administered over 21 months is now recommended by WHO beginning at 5 months of age^9^. A second vaccine based on the PfCSP, R21 had results comparable to RTS,S/AS01 in a Phase 2 trial, and is now in Phase 3^10^.

PfSPZ Vaccine, comprised of radiation-attenuated, aseptic, purified, cryopreserved Pf sporozoites (SPZ), expresses thousands of proteins and induces protective immunity against the early, clinically silent stages of malaria through antibodies and T cells^11–21^. It has demonstrated superior vaccine efficacy (VE) as compared to subunit vaccines, inducing greater than 90% VE against controlled human malaria infection (CHMI) in the US and Africa, and significant VE against naturally transmitted malaria in African adults^11–18^. However, VE against parasites that are genetically and thus antigenically different (heterologous) from the vaccine strain has been lower than VE against homologous parasites. For example, VEs of 92 and 70% were demonstrated against homologous CHMI performed 3 and 24 weeks after vaccination, respectively, using the same West African parasite as in the vaccine (PfNF54), but only 80 and 10%, respectively, against heterologous CHMI conducted in parallel using the divergent Pf7G8 parasite from Brazil^14^. Pf7G8 has now been shown to be more distant from PfNF54, the vaccine strain, than 704 Pf isolates from West, East, and Central Africa^19^. Furthermore, when the same regimen of PfSPZ Vaccine was assessed against CHMI 6 months after the last dose of PfSPZ Vaccine in the US and against intense transmission of heterogeneous Pf in the field in Mali during the 6 months after the last dose of vaccine, VE was as good, if not better, in the field than against CHMI^14,16,18,19,22^. Thus, heterologous CHMI with Pf7G8 provides a rigorous assessment of the VE of PfSPZ Vaccine. Strain-transcending protection is required if the vaccine is to protect against the diverse parasites encountered in the field, and we surmised that more effective cross-strain protection could be achieved through an improved vaccination regimen.

This study was designed to identify a PfSPZ Vaccine regimen that could move to late Phase 2 and pivotal Phase 3 clinical trials. The goal was a well tolerated, safe vaccination regimen that could be administered within 28 days and had at least 75% VE for at least 9 weeks (a time period longer than >80% of all travel to Africa)^23^ against CHMI with heterologous Pf parasites that are more distant from the vaccine strain than essentially all Pf parasites in Africa, a criterion met by Pf7G8^19,24^. Because PfSPZ of PfSPZ Vaccine develop only partially in the liver and do not replicate, there is less in vivo amplification of the immunogen than with other live vaccines; for this reason, we selected two doses one week apart as the prime (multidose priming)^22^. Moreover, since the vaccine induces antibody responses targeting the sporozoite, we were concerned this could diminish the effectiveness of a delayed boost, and therefore selected a short, three-week prime-boost gap to dodge the peak antibody response. We hoped these two features of the immunization regimen would increase vaccine potency and improve cross-strain protection. Finally, we felt that the practicality of a four-week regimen would support timely travel preparations, seasonal malaria prophylaxis in children, protection of newly pregnant women and efficient administration to entire populations in mass vaccination programs.

## Results

### Trial participants

**Fig. 1:**
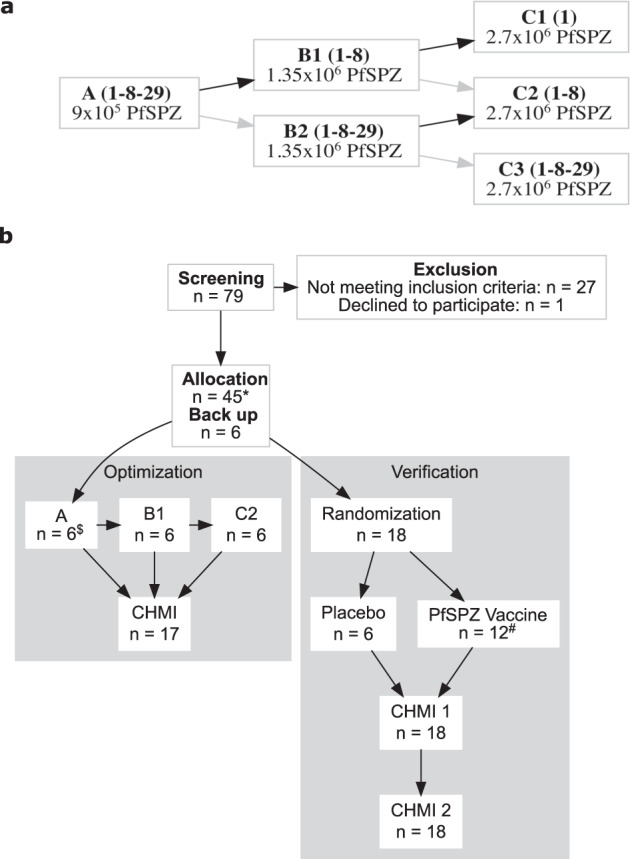
Study flow Chart. **a** *Optimization* of PfSPZ Vaccine regimen and (**b**) *Verification* of PfSPZ Vaccine regimen. *9 of the 45 subjects were allocated to a 7G8 safety analysis that will be published elsewhere. ^$^One volunteer received only the first vaccination. ^#^One volunteer received the 2nd vaccination on day 15 instead of day 8.

**Fig. 2:**
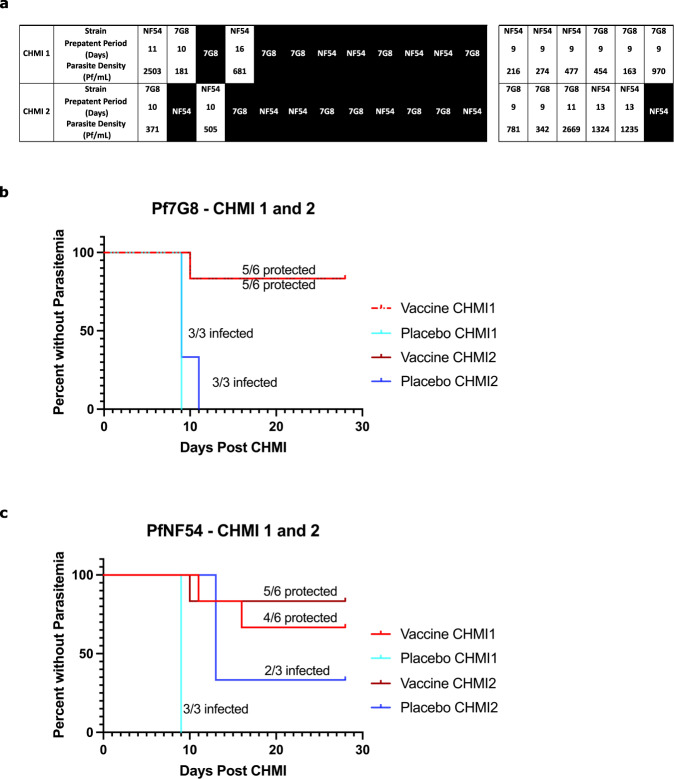
Results of CHMIs to assess VE during *Verification*. **a** First CHMI at 3 weeks (top row) and second CHMI at 9–10 weeks (bottom row) of the individual volunteers. The black indicates no parasitemia (protected); the white represents parasitaemia (not protected). **b** Kaplan-Meier plots for heterologous CHMIs with Pf7G8. Five of 6 vaccinees were protected at 3 weeks and at 9–10 weeks. Six of 6 CHMIs with PfSPZ Challenge (7G8) in the controls resulted in parasitemia (infected). **c** Kaplan-Meier plots for homologous CHMIs with PfNF54. Four of 6 vaccinees were protected at 3 weeks and 5/6 were protected at 9–10 weeks. Five of 6 CHMIs with PfSPZ Challenge (NF54) in the controls resulted in parasitemia (infected).

From 22 September 2016 to 1 March 2018, 45 volunteers were enrolled sequentially. Nine of the 45 were allocated to a study of the safety and infectivity of Sanaria® PfSPZ Challenge (7G8) that will be reported separately. The remaining 36 were randomized into two cohorts. In part 1 (*optimization* phase) an optimal regimen was identified. In part 2 (*verification* phase) the optimal regimen was assessed prospectively to verify its efficacy. During *Optimization* all volunteers received at least one dose of PfSPZ Vaccine and 17/18 completed their schedule and underwent CHMI (Fig. 1b). One volunteer withdrew from further vaccinations and CHMI after the first dose of vaccine for personal reasons and subsequently was followed to monitor safety. All 12 vaccinees and 6 controls in the *verification* pha﻿se completed all inoculations: three vaccinations and two CHMIs. Vaccinations were done with PfSPZ Vaccine or normal saline (placebo), CHMI with PfSPZ Challenge (NF54) and PfSPZ Challenge (7G8). CHMIs were done using a cross-over design with randomly allocated sequences of NF54-7G8 and 7G8-NF54 (Figs. 1b and 2a). One volunteer allocated to PfSPZ Vaccine received the second vaccination on day 15 instead of day 8 (Fig. 1b). The third vaccination was administered and CHMIs were conducted according to schedule. Demographic characteristics were similar between the groups of healthy, malaria-naïve, young adults (Table 1).

**Table 1:** Demographics of trial participants.

Trial stage	Arm	Sex (f/m)^a^	Age (years)^b^	Height (cm)^b^	Weight (kg)^b^	BMI (kg/m^2^)^b^
*Optimization*	A	5/1	23.4 (21.2–27)	166 (161–178)	60 (51–75)	20.6 (18.3–27.5)
	B1	5/1	27.7 (26.6–29)	169 (163–187)	67 (56–87)	22.9 (19.4–30.8)
	C2	3/3	25.1 (21.8–42.4)	173 (163–179)	66 (55–83)	22.1 (20.7–27.4)
*Verification*	PfSPZ Vaccine	4/8	26.9 (20.2–32.5)	179 (160–194)	74 (52–103)	22.3 (18–30.1)
	Placebo	4/2	24.6 (20.6–26.5)	172 (152–190)	68.5 (55–100)	23.7 (21.1–27.7)

^a^count; ^b^median (range).

### Safety and tolerability

Immunization with PfSPZ Vaccine was generally well tolerated and safe (Table 2, Supplementary Tables 1–4).

**Table 2:** **a** Solicited adverse events during immunization during *Verification*. **b** Unsolicited adverse events during immunization considered to be related to immunization during *Verification*.

Preferred term*	Severity grade	PfSPZ Vaccine (*n* = 12)			Placebo (*n* = 6)			*P* value* (vaccine vs. placebo)
		AE (*n*)	Volunteers with AE (*n*)	Volunteers with AE (%)	AE (*n*)	Volunteers with AE (*n*)	Volunteers with AE (%)	
Chills	1	3	3	25	0	0	0	0.52
Diarrhea	1	1	1	8.3	0	0	0	1.0
Dizziness	1	1	1	8.3	2	2	33.3	1.0
Fatigue	1	11	8	66.7	2	2	33.3	0.32
Headache	1	11	7	58.3	2	2	33.3	0.62
	2	3	2	16.7	0	0	0	0.53
Myalgia	1	4	4	33.3	0	0	0	0.25
Pyrexia	1	3	3	25	0	0	0	0.55

MedDRA Preferred term	Severity grade	PfSPZ Vaccine (*n* = 12)		Placebo (*n* = 6)		*P* value* (vaccine vs. placebo)
		Volunteers with AE (*n*)	Volunteers with AE (%)	Volunteers with AE (*n*)	Volunteers with AE (%)	
Any AE	1	3	25	2	33.3	1
	2	3	25	2	33.3	1
Nervous system disorders	1	2	16.6	2	33.3	0.57
	2	2	16.6	0	0	0.53
Psychiatric disorders	1	1	8.8	0	0	1
	2	1	8.8	0	0	1
Gastrointestinal disorders	1	1	8.8	0	0	1
	2	0	0	1	16.6	0.33
Musculoskeletal and connective tissue disorders	1	0	0	0	0	N/A
	2	1	8.8	0	0	1
General disorders and administration site conditions	1	3	25	0	0	0.51
	2	0	0	0	0	N/A
Infections and infestations	1	1	8.8	1	8.8	1
	2	1	8.8	0	0	1
Respiratory, thoracic and mediastinal disorders	1	0	0	0	0	N/A
	2	1	8.8	0	0	1

*2-tailed Fisher’s exact test for # volunteers with AEs.

#### Optimization Phase

The results of safety assessment during the *optimization* phase that did not include controls are described in Supplementary Tables 1–3. At the highest dose of PfSPZ Vaccine (2.7 × 10^6^ PfSPZ), one volunteer reported a constellation of grade 3 symptoms the night following the second vaccination (vomiting, chills, hyperhidrosis, myalgia, fatigue) considered related to the vaccination, that subsided over the next 2 days and did not reoccur after CHMI. This volunteer was protected at CHMI. A second participant experienced fever to 39.2 °C two days after the 3^rd^ dose of 9.0 × 10^5^ PfSPZ accompanied by mild fatigue that resolved by the next day. Two subjects had Grade 3 lymphopenia and one subject had Grade 3 decreased glucose.

#### Verification phase

There was no significant difference between vaccinees and controls in solicited or unsolicited adverse events (AEs) during the immunization period (Table 2). During the immunization period, there were no Grade 3 solicited AEs, no Grade 3 immunization-related unsolicited AEs (Table 2), and no Grade 3 laboratory abnormalities (Supplementary Table 3). One vaccinee did have Grade 3 elevations of systolic blood pressure that was considered to be unrelated to immunization. There was one serious adverse event (SAE) in a placebo recipient who had a sports accident 126 days following the first vaccination. This SAE was not considered related to the study interventions. Lymphopenia was more commonly observed in the vaccine group (10 of 12 subjects, Supplementary Table 3) than in the placebo controls (0/6, p = 0.0015, Fisher’s Exact test). Most episodes occurred 1 day after immunization and resolved by the next measurement 6 days later.

#### CHMI

One participant in the *optimization* phase experienced grade 3 headache and fatigue with CHMI, corresponding to the time of Pf parasitemia. During the *verfication* phase PfSPZ Challenge (NF54) and PfSPZ Challenge (7G8) were safe and caused only mild or moderate symptoms as a result (Supplementary Table 4). The patterns of symptoms were similar for NF54 and 7G8 (Supplementary Table 4). The incidence of headache, the most frequently encountered moderate to severe AE, was higher, but not significantly, in infected and uninfected participants in both phases (5/21 with parasitemia vs. 3/32 without parasitemia, *p* = 0.24, Fisher’s exact test) (Supplementary Table 4). Grade 3 lymphopenia, associated with parasitemia, occurred after 2/6 Pf7G8 and 1/6 PfNF54 CHMIs.

### Vaccine efficacy

#### Optimization phase

All subjects excluding the one drop-out from the first group underwent homologous CHMI with PfSPZ Challenge (NF54) 3 weeks after the last dose of vaccine. The first regimen, 9.0 × 10^5^ PfSPZ on days 1, 8 and 29 (Group A), met pre-specified VE criteria: none of the 5 subjects undergoing CHMI developed parasitaemia (1-risk ratio x 100 = 100%). The decision tree then directed study flow to a two-dose group receiving 1.35 × 10^6^ PfSPZ on days 1 and 8 (Group B1), which is the same total dose of PfSPZ as the first regimen (2.7 × 10^6^ PfSPZ). This tested the hypothesis that only total dose mattered and that two injections would be as efficacious as three. All six subjects underwent homologous CHMI three weeks later and 2/6 developed parasitaemia (1-risk ratio x 100% = 67%), a VE not sufficient to progress to a single dose regimen. The decision tree directed the third group to retain a 2-dose regimen but with an increased dose per injection, in an effort to preserve the simpler 2-dose approach. However, doubling the dose from 1.35 × 10^6^ to 2.7 × 10^6^ PfSPZ (Group C2) did not improve VE as 3/6 developed parasitaemia (1-risk ratio x 100% = 50%) (Supplementary Fig. 2). In summary, only the initial, 3-dose regimen (9.0 × 10^5^ PfSPZ on days 1, 8 and 29) met VE criteria. This regimen was therefore selected for transition to the *verification* phase as the shortest sufficiently efficacious regimen.

#### Verification phase

Twelve participants were administered PfSPZ Vaccine and 6 normal saline placebo according to the 3-dose regimen, and all underwent CHMI 21 days (3 weeks) later; within each arm, half the participants were randomly and blindly assigned to Pf7G8 and half to PfNF54. Forty-two to 47 days later, at 63–68 days after the last dose of vaccine (9–10 weeks), all subjects underwent a second CHMI with the Pf strain reversed (double cross-over design).

#### CHMI with PfSPZ Challenge (7G8)

Among vaccinees who underwent CHMI with PfSPZ Challenge (7G8), 2/12 developed parasitemia (1/6 after the 3 week CHMI and 1/6 after the 9–10 week CHMI) and 6/6 normal saline placebo control subjects developed parasitemia (3/3 after the 3 week CHMI and 3/3 after the 9–10 week CHMI) (Fig. 2). The controls developed parasitemia on day 9 after the first CHMI (3 of 3) or on day 9 (2 of 3) or day 11 (1 of 3) after the second CHMI. One vaccinee developed parasitemia on day 10 after CHMI 1 and one vaccinee developed parasitemia on day 10 after CHMI 2. Crude VE (1-risk ratio) against heterologous CHMI was 83% after each CHMI.

#### CHMI with PfSPZ Challenge (NF54)

Among vaccinees who underwent CHMI with PfSPZ Challenge (NF54), 3/12 developed parasitemia (2/6 after the 3 week CHMI and 1/6 after the 9–10 week CHMI) and 5/6 normal saline placebo control subjects developed parasitemia (3/3 after the 3 week CHMI and 2/3 after the 9–10 week CHMI) (Fig. 2). Parasitemia developed in the three controls on day 9 after the first CHMI and in the two positive controls on day 13 after the second CHMI. The three vaccinees who were not protected developed parasitemia on days 11 and 16 (CHMI 1), and 10 (CHMI 2). Crude VE (1-risk ratio) against homologous CHMI was 67% after the first CHMI and 75% after the second CHMI.

Our primary calculation of VE was by proportional analysis using generalized linear regression with a logit link function (logistic regression). Overall VE compared to placebo was 78% (95% predictive interval: 57%; 92%). VEs against PfSPZ Challenge (NF54) and PfSPZ Challenge (7G8) were 77% (95%PI: 50%; 95%) and 79% (95%PI: 54%; 95%), respectively. The prediction interval estimates the uncertainty about future vaccinee response and is therefore larger than a confidence or credible interval. Sensitivity analyses with different priors and frequentist methods were performed and they had no substantial effect on VE estimates.

One volunteer was not protected in either CHMI, three developed parasitaemia once (two in the first and one in the second CHMI) and eight were protected in both CHMIs (Fig. 2a), including the volunteer who received the second vaccination on day 15 (see above).

Repeat CHMI of placebo controls with first PfSPZ (NF54) and second PfSPZ (7G8), or vice versa, resulted in infection rates of 6/6 in the first CHMI and 5/6 in the second CHMI. One placebo recipient developed parasitaemia following first CHMI with Pf7G8 but not following second CHMI with PfNF54. For Pf7G8 the pre-patent periods were 9,9,9 days in the first CHMI and 9,9,11 days in the second CHMI. For PfNF54 they were 9,9,9 days in the first CHMI and 13,13, and negative in the second CHMI (Fig. 2). An initial infection with PfNF54 did not impact a subsequent infection with Pf7G8, but an initial infection with Pf7G8 was associated with a delay in the onset of infection with PfNF54 in the second CHMI.

### Immunogenicity

In the *optimization* phase, all volunteers developed antibodies to PfCSP by the day before CHMI at 3 weeks (Supplementary Fig. 1 and Supplementary Table 6). PfSPZ Vaccine at a dose of 1.35 × 10^6^ on day 1 and 8 induced lower antibody concentrations compared to the other regimens (Supplementary Fig. 1 and Supplementary Table 6).

**Fig. 3:**
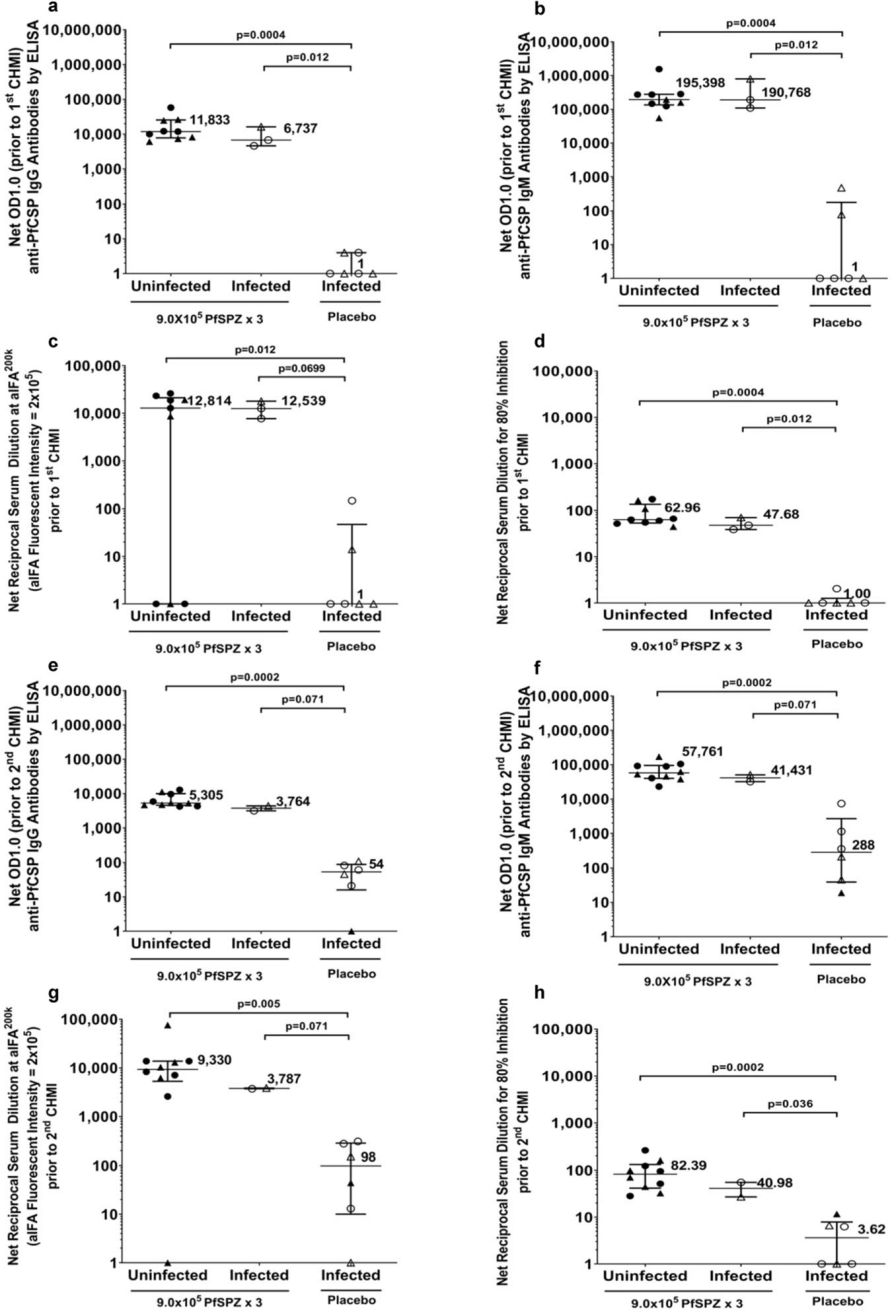
Antibodies to PfCSP and PfSPZ and functional activity of sera against PfSPZ. **a**, **b** Median and interquartile range of net OD 1.0 for IgG and IgM antibodies to PfCSP by ELISA one day prior to CHMI in malaria-naïve adults who were uninfected (protected) and infected during CHMI administered 3 weeks after the 3^rd^ dose (CHMI 1). **c, d** Median and interquartile range of net IgG antibodies to PfSPZ by aIFA and net inhibition of PfSPZ invasion of hepatocytes by aISI one day prior to CHMI in malaria-naïve adults who were uninfected (protected) and infected during CHMI administered 3 weeks after the 3^rd^ dose (CHMI 1). **e, f** Median and interquartile range of net OD 1.0 for IgG and IgM antibodies to PfCSP by ELISA the day prior to CHMI in adults who were uninfected (protected) and infected during CHMI administered 9–10 weeks after the 3^rd^ dose (CHMI 2). **g, h** Median and interquartile range of net IgG antibodies to PfSPZ by aIFA and net inhibition of PfSPZ invasion of hepatocytes by aISI one day prior to CHMI in adults who were uninfected (protected) and infected during CHMI administered 9–10 weeks after the 3^rd^ dose (CHMI 2). P values were calculated by Wilcoxon-Mann-Whitney test. For each panel, filled triangles are uninfected subjects and open triangles are infected subjects who received heterologous CHMI with PfSPZ Challenge (7G8) and filled circles are uninfected subjects and open circles are infected subjects who received homologous CHMI with PfSPZ Challenge (NF54).

In the *verification* phase, one day prior to CHMI 1, as compared to pre-immunization, sera from all vaccinees had an increased level of IgG and IgM antibodies to PfCSP by enzyme-linked immunosorbent assay (ELISA) and increased inhibition of sporozoite invasion of hepatocytes by inhibition of sporozoite invasion assay (aISI) (Fig. 3a, b, d and Supplementary Tables 7 and 8). However, only 9/12 vaccinees developed IgG antibodies to PfSPZ detected by semi-automated PfSPZ immunofluorescence assay (aIFA) (Fig. 3c and Supplementary Tables 7 and 8). All three of the vaccinees who did not seroconvert in the aIFA were protected from the first CHMI (Fig. 3c). None of the normal saline placebo recipients met the criteria for an increase in activity in any of the three assays. One day prior to CHMI 2, two of the three subjects who had not met the criteria for seroconversion in the aIFA at the time of the first CHMI met criteria for seroconversion. The one non-converter was protected in CHMI 2. (Fig. 3g). Several of the placebo controls met criteria for sero-conversion for IgM antibodies to PfCSP (2/6) and PfSPZ by aIFA (3/6), albeit at levels of antibodies much lower than in the vaccinees (Fig. 3f, g); none developed significant activity in aISI assay. There was no significant difference between protected and unprotected vaccinees for any of the assays at CHMI 1 or 2. However, since there were so few unprotected vaccinees, there was little power to show such differences.

## Discussion

The radiation-attenuated PfSPZ Vaccine is moving forward in clinical development based on reproducible VE against CHMI^13,14,17,25^ and in the field^16,18^ and on excellent tolerability and safety^16,18,20,21,26,27^. More than 6000 doses of PfSPZ of PfSPZ Vaccine containg 5.2 billion PfSPZ have been administered to > 2000 subjects aged 5 months to 61 years in 20 clinical trials in Africa, Europe, and the US without documenting a breakthrough infection. In double blind placebo-controlled trials, including 12 conducted in Africa, there have been no significant differences in adverse events or laboratory abnormalities between vaccinees and normal saline controls^16,18,20,21,26–29^. The near absence of reactogenicity of PfSPZ Vaccine was confirmed in a recent trial completed in Kenyan infants who were administered three doses of 4.5 × 10^5^, 9.0 × 10^5^, or 1.8 × 10^6^ PfSPZ, the latter twice the dose administered to adults in the current study^27,28^.

We report two major contributions to development of PfSPZ Vaccine for prevention of Pf malaria. We have achieved greater than 75% VE for at least 9 weeks against CHMI with Pf7G8, a Pf parasite that is more distant at the genome, proteome, and CD8 T cell immunome levels from the NF54 strain of Pf in PfSPZ Vaccine than any of 704 Pf isolates of Pf from East, West, and Central Africa^19^. We have also reported previously that it was more difficult to protect US adults against CHMI with Pf7G8 at 6 months than Malian adults against Pf naturally acquired in Africa over 6 months of surveillance^14,16,18,19,22^, indicating that CHMI in malaria-naïve adults with Pf7G8 was as, if not more, stringent a test of VE than natural exposure of adults in Africa. This finding suggests that PfSPZ Vaccine protection against Pf7G8 CHMI should translate to protection across the African continent as long as sufficiently robust immune responses can be induced.

Second, we have accomplished this with a 3-dose, 4-week immunization regimen. Compared to the 8 to 24 week regimens often used for vaccines including the early field trials of PfSPZ Vaccine^16–18,20,21,26,28^, this compact regimen will facilitate compliance and support the need for rapid immunization prior to travel and in vaccination programs. We tried to reduce the numbers of doses to two and the time to immunize to 7 days, but this did achieve the same level of VE. We doubt that further condensation of the regimen will provide comparable VE.

The key component of the down-selected regimen may be the two sequential doses in the first week – multidose priming. Prior work indicates that this approach enhances the VE of PfSPZ Vaccine compared to single dose priming^22,29^. Because the PfSPZ in PfSPZ Vaccine do not replicate, administering two or more doses in a short period of time may better mimic the typical live attenuated vaccines used against many pathogens, which generally replicate over the course of several days before containment by nascent immune responses.

In the *optimization phase* of the study, we started with a contracted regimen of 3 doses of 9.0 × 10^5^ PfSPZ. We protected 100% (5/5) of vaccinees against PfNF54 CHMI at 3 weeks after the last dose. Further contraction to a 1-week, 2-dose regimen decreased VE, despite the same or higher total PfSPZ dose (67 and 50% were protected, respectively). The failure of dose escalation (from 1.35 × 10^6^ to 2.7 × 10^6^ PfSPZ) to increase VE is consistent with prior experience in Africa where escalation from 9.0 × 10^5^ to 1.8 × 10^6^ PfSPZ led to a reduction of VE rather than improvement^17^. As we have described, this may be due to high dose tolerance/suppression of T cell responses^17^.

In the *verification phase* of the study we further tested the 3-dose regimen of 9.0 × 10^5^ PfSPZ on days 1, 8, and 29. At 3 weeks after last immunizing dose half the subjects underwent CHMI with PfSPZ Challenge (NF54) and half with PfSPZ Challenge (7G8). At 9–10 weeks, the subjects who had received CHMI with PfSPZ Challenge (NF54) underwent CHMI with PfSPZ Challenge (7G8) and vice versa. Among those who underwent CHMI with Pf7G8, 6/6 controls developed parasitaemia, and 5/6 (83%) vaccinees were protected at first CHMI and 5/6 (83%) vaccinees were protected at second CHMI. In a prior study, when 9.0 × 10^5^ PfSPZ was administered 3 times at 8-week intervals, there was 20% VE against Pf7G8 CHMI at 12 weeks^22^. These data indicate that the contracted regimen is certainly as good as the longer regimen, and likely superior.

As in previous studies, PfSPZ Vaccine was safe and well tolerated when given at doses up to 2.7 × 10^6^ PfSPZ, although one volunteer reported a transient, self-limiting systemic reaction the evening after receiving the second dose of 2.7 × 10^6^ PfSPZ. Reactogenicity may have resulted from the short time between first and second doses – a seven-day interval vs. the eight-week interval used between doses in the other studies of this dose. Self-limiting systemic reactions were reported in one individual in another trial^21^. An interesting observation was the occurrence of short, asymptomatic episodes of lymphopenia on the day following vaccination. We selected this timepoint for safety assessments primarily to check for possible signs of liver injury due to the high number of PfSPZ. No evidence of liver injury was detected. Only few vaccine studies report laboratory results shortly following vaccination. Interestingly, in one systematic study on yellow fever vaccine transient drops in lymphocyte counts were also observed^30^.

Vaccination with PfSPZ Vaccine induced antibodies against PfSPZ and the main surface antigen of PfSPZ, PfCSP. These antibodies prevented sporozoite invasion of hepatocytes in vitro. However, in contrast to what we have seen in some previous studies of PfSPZ in malaria naïve adults^31^ and in field studies in Africa^16,18,28^, there was no significant association between levels of response in any of the three antibody assays we used, and protection from Pf infection. This may be due to the minimal power to detect significant differences in this small study with only a few unprotected vaccinees, since IgG antibodies to PfCSP and inhibitory activity in the aISI assay were higher in protected vs unprotected vaccinees (Fig. 3). The results of this study were consistent with the results of other studies of PfSPZ Vaccine in that IgM antibodies to PfCSP were apparently higher than IgG antibodies to PfCSP, and were still quite high 9 weeks after the last dose of vaccine^28^. It seems that the class switching from IgM to IgG classically described for responses to viral and bacterial infections, and vaccines against these infections, does not apply to the whole parasites that comprise PfSPZ^32,33^. The lack of association of antibody results with protection was consistent with the generally accepted concept that the protection by PfSPZ Vaccine is mediated by tissue-resident T cells in the liver, especially CD8 + T cells, that eliminate Pf-infected hepatocytes directly through cytolysis or indirectly by secretion of interferon gamma, which induces infected hepatocytes to produce nitric oxide that eliminates the infected hepatocytes or through a cytokine cascade that leads to the same result^12,13,25,34–37^. The PfSPZ in our vaccine express ~1,000 proteins and unfortunately, which and how many of those are involved in the protective efficacy of PfSPZ Vaccine are not known^38^.

In summary, the identified regimen was safe and well-tolerated and protected >75% of vaccinees for at least 9 weeks against CHMI with a heterologous Pf parasite. Based on the results of the present trial, PfSPZ Vaccine was assessed in a Phase 2 trial of women of child-bearing potential in Mali (NCT03989102) and has shown unprecedented protective efficacy against Pf infection and clinical malaria during two malaria transmission seasons over 18 months^39^, is being assessed in a Phase 2 trial in non-immunes (NCT04966871) and in a Phase 2 trial in 6-10-year-old children in Mali (NCT04940130).

## Methods

### Trial participants

We recruited healthy, malaria-naïve volunteers, aged 18–45 years at the clinical trial facility of the Institute of Tropical Medicine, Tübingen University Hospital, Germany. Following written informed consent, volunteers underwent medical and laboratory examinations to minimize risks of study participation. Details are given in the accompanying study protocol (Supplement).

### Trial design, randomization and treatments

The trial had 2 parts, the first an open-label regimen *optimization* phase and the second a randomized, double-blind, normal saline placebo controlled *verification* phase. The aim of the *optimization* phase was to identify the shortest, well tolerated and efficacious immunization regimen by algorithmically varying the number and size of PfSPZ doses, measuring VE for each regimen using homologous CHMI 3 weeks after vaccination. An adaptive design was used, starting with three doses and navigating through a pre-determined decision tree adjusting dose size and aiming for two- and potentially one-dose regimens depending on whether VE criteria were met (Fig. 1a). The aim of the *verification* phase was to assess VE of the optimal regimen down-selected from part 1 against both homologous and heterologous CHMI performed 3 and 9–10 weeks after immunization. The blinding and inclusion of normal saline controls in the *verification* phase also allowed the collection of robust data on tolerabity and safety.

#### Optimization

The immunization regimen was optimized using the pre-specified decision tree with groups of 6 volunteers, starting with 9 × 10^5^ PfSPZ of PfSPZ Vaccine on day 1, 8 and 29. PfSPZ Vaccine was administered by direct venous inoculation (DVI). The maximum single PfSPZ dose was set at 2.7 × 10^6^ PfSPZ. Decisions on either regimen contraction or dose escalation were based on achieving at least 5/6 protected volunteers (Fig. 1a).

#### Verification

To verify the optimized regimen, volunteers were allocated randomly and blindly to either the optimized PfSPZ Vaccine regimen (*n* = 12) or to placebo (*n* = 6). Assignment to PfSPZ Challenge (NF54 and 7G8) at 3 weeks was also done by random allocation and the other strain was then to be administered at 9–10 weeks (double cross-over design); all subjects underwent CHMI twice, first with either the homologous or heterologous Pf parasites and second with the Pf parasites they did not receive in the first CHMI. In the event that a one- or two-dose regimen progressed to the *verification* phase, a second vaccine group (*n* = 12 vaccinees and *n* = 6 controls) using a three-dose regimen (day 1, 8, 29) at the same dose-level (maximum regimen) was to be added.

#### CHMI

Homologous CHMI was performed using the standard 100% infectious dose of 3.2 × 10^4^ PfSPZ of PfSPZ Challenge (NF54)^40–43^ and heterologous CHMI using the same dose of PfSPZ Challenge (7G8)^31,44,45^, all doses administered by DVI. No infectivity controls were enrolled for the homologous CHMIs performed during *Optimization*. This was because all 79 malaria-naive control subjects who had previously undergone CHMI using PfSPZ Challenge (NF54) in 14 different trials at six different institution in the US, Germany and Gabon had become positive, indicating that the 3.2 × 10^4^ PfSPZ dose is 100% infectious in malaria-naive subjects^31,41–44,46–49^. In addition, stability data for PfSPZ Challenge provided by Sanaria, which like other PfSPZ products is stored in liquid nitrogen vapor phase, indicated essentially no loss in viability over years of testing (data not shown). We were therefore confident that all vaccinees undergoing CHMI in part 1 would receive infectious doses of PfSPZ, and that estimates of VE would be accurate. However, infectivity controls were included in the *verification* phase. This is because *Verification* included repeat CHMI, meaning that research subjects were no longer malaria-naive at the time of the second CHMI. Because 3.2 × 10^4^ PfSPZ of PfSPZ Challenge (NF54) is not always 100% infectious in malaria-exposed individuals^47,50,51^, the six placebo recipients were used as infectivity controls for both the first and second CHMI specifically to measure the infectivity of the second CHMI.

#### Tolerability and safety

During immunization, volunteers were monitored on-site and regularly contacted by telephone. At all visits and calls AEs were collected and coded using the Medical Dictionary for Regulatory Activities (MedDRA Version 22). AEs were collected from the day of first immunization until the day of CHMI at 3 weeks to address the primary safety outcomes - the frequency of related grade 3 (severe) and serious AEs. Samples for hematological and biochemical investigations were taken before, one and seven days after each vaccination and before and during CHMIs. Samples for immunological evaluations were taken before the first and 14 days after the third vaccination as well as before, seven and 14 days after each CHMI and on the day of treatment in case parasitemia developed. Additional safety and serum samples were taken 28, 56 and 105 days after the last CHMI.

#### Malaria diagnosis and treatment

Volunteers were assessed at least daily from day 6 following initiation of CHMI and antimalarial treatment was started when reverse transcription quantitative polymerase chain reaction (RT-qPCR) was positive in at least three measurements, each >12 h apart and one of which had to be >100 parasites/mL, or if any thick blood smear became positive. The prepatent period was defined as the time from CHMI to the first RT-qPCR with >100 parasites/mL. Follow-up visits beyond day 28 post-CHMI were done monthly for 4–6 months. All malaria cases during CHMI were treated with atovaquone-proguanil or artemether-lumefantrine.

### Randomization and masking

During *Verification* the allocation to intervention was nested within the allocation to immunization. The computer-generated randomizations were done by an independent party on the eve of the first immunization day and the first CHMI, respectively. Volunteers and the clinical, immunology and diagnostic teams were blinded. Unblinded members of the syringe preparation team had no other role in the trial. Allocation was concealed until after the second CHMI. Syringes of PfSPZ Vaccine and placebo were of the same type. PfSPZ Vaccine and placebo have exactly the same appearance.

### Statistical analysis

Tolerability and safety data were described and tabulated without formal hypothesis testing. VE, assessed by repeat CHMI, was modelled using Bayesian generalized linear regression and reported with the respective prediction intervals. The model included an intercept and intervention. To calculate strain-specific VE, strain was included as an additional variable. The priors were weakly informative, based on results of previous studies and the *optimization phase* (intervention).

### Parasitological and immunological investigations

Quantitative thick blood smear^52^ and ultrasensitive RT-qPCR^43^ were done as described previously with lower limits of detection of 5 parasites/µL and the nucleic-acid-equivalent of 6 parasites/mL, respectively. Immunogenicity was assessed to Pf circumsporozite protein (CSP) by ELISA, and to PfSPZ by automated immunofluorescence assay (aIFA) and automated inhibition of sporozoite invasion assay (aISI) before first vaccination, 14 days following the last dose of vaccine and the day before CHMI as described^43^.

### Trial oversight

The trial was approved and overseen by the Paul Ehrlich Institute, the U.S. Food and Drug Administration under an Investigational New Drug application and the Ethics Committee of the Medical Faculty and the University Clinics of the University of Tübingen (Number 023/2016AMG1). An independent monitoring committee oversaw safety aspects of the trial and approved transition from *Optimization* to *Verification*. The trial is registered with ClinicalTrials.gov, NCT02704533.

### Role of the funding source

The clinical trial was funded by the Deutsches Zentrum für Infektionsforschung (DZIF). Manufacture of PfSPZ Vaccine, PfSPZ Challenge (NF54) and PfSPZ Challenge (7G8) was funded in part by the National Institute of Allergy and Infectious Diseases of the National Institutes of Health through SBIR award numbers 5R44AI058375 and 5R44AI055229. Both funders had no role in planning, conduct, analysis and publication of the results.

### Reporting Summary

Further information on research design is available in the Nature Research Reporting Summary linked to this article.

## Supplementary information

Supplementary Information

REPORTING SUMMARY

## Data Availability

The data supporting the findings of this study are available within the Article and its Supplementary Information and from the corresponding author upon reasonable request.
