# Supplementary material for: A PfSPZ vaccine immunization regimen equally protective against homologous and heterologous controlled human malaria infection
Source: NPJ Vaccines. 2022 Aug 23;7:100. doi: 10.1038/s41541-022-00510-z (PMC9396563; doi:10.1038/s41541-022-00510-z)
Supplement: Supplementary file 1 — Supplementary Information [file 41541_2022_510_MOESM1_ESM.pdf]

**Supplemental Information**

# A PfSPZ Vaccine Immunization Regimen Equally Protective Against Homologous and Heterologous Controlled Human Malaria Infection

Benjamin Mordmüller et al.

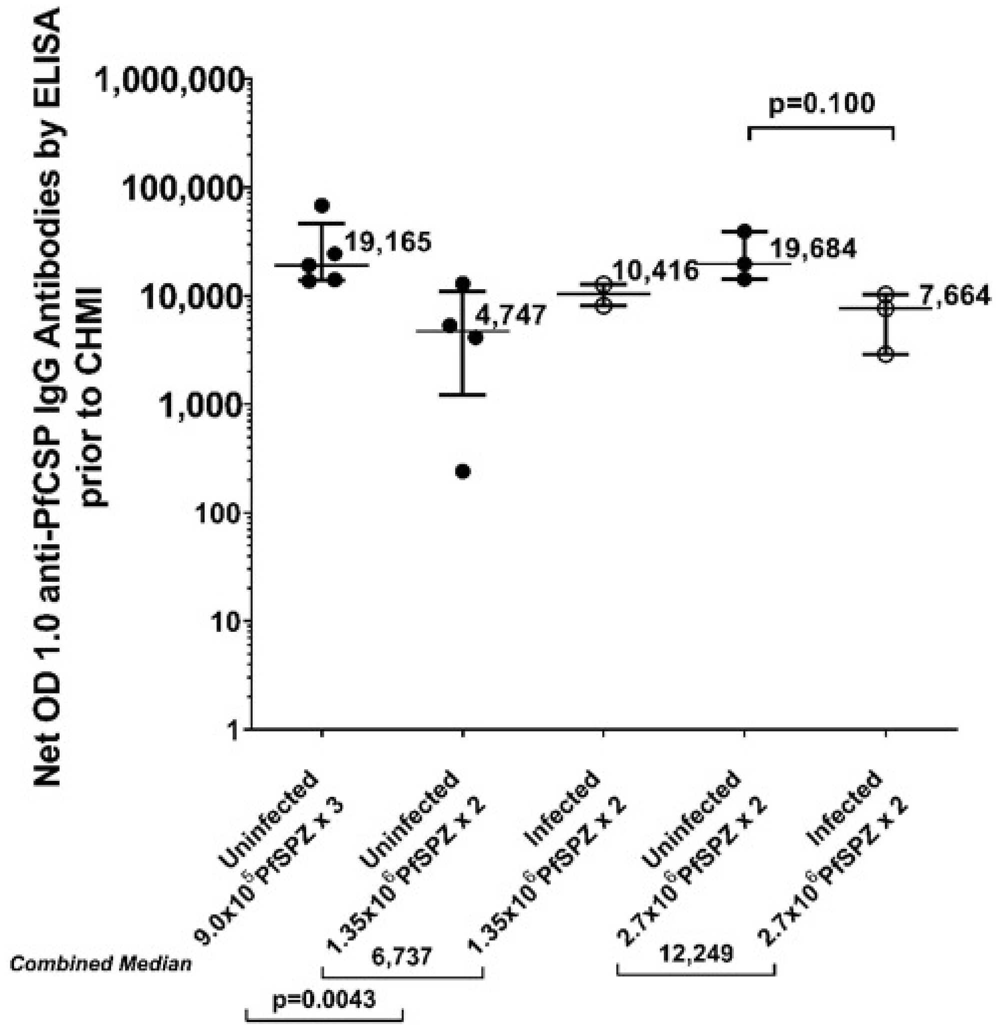


**Supplementary Figure 1.** ***Optimization phase*****:** Median and interquartile range of net OD 1.0 for IgG antibodies to PfCSP one day prior to CHMI of PfSPZ Vaccine in malaria-naïve adults who were uninfected (protected) and infected during CHMI administered 3 weeks after the last dose. P value calculated by Wilcoxon-Mann-Whitney test. For each panel, filled circles are uninfected subjects and open circles are infected subjects who received homologous CHMI with PfSPZ Challenge (NF54).

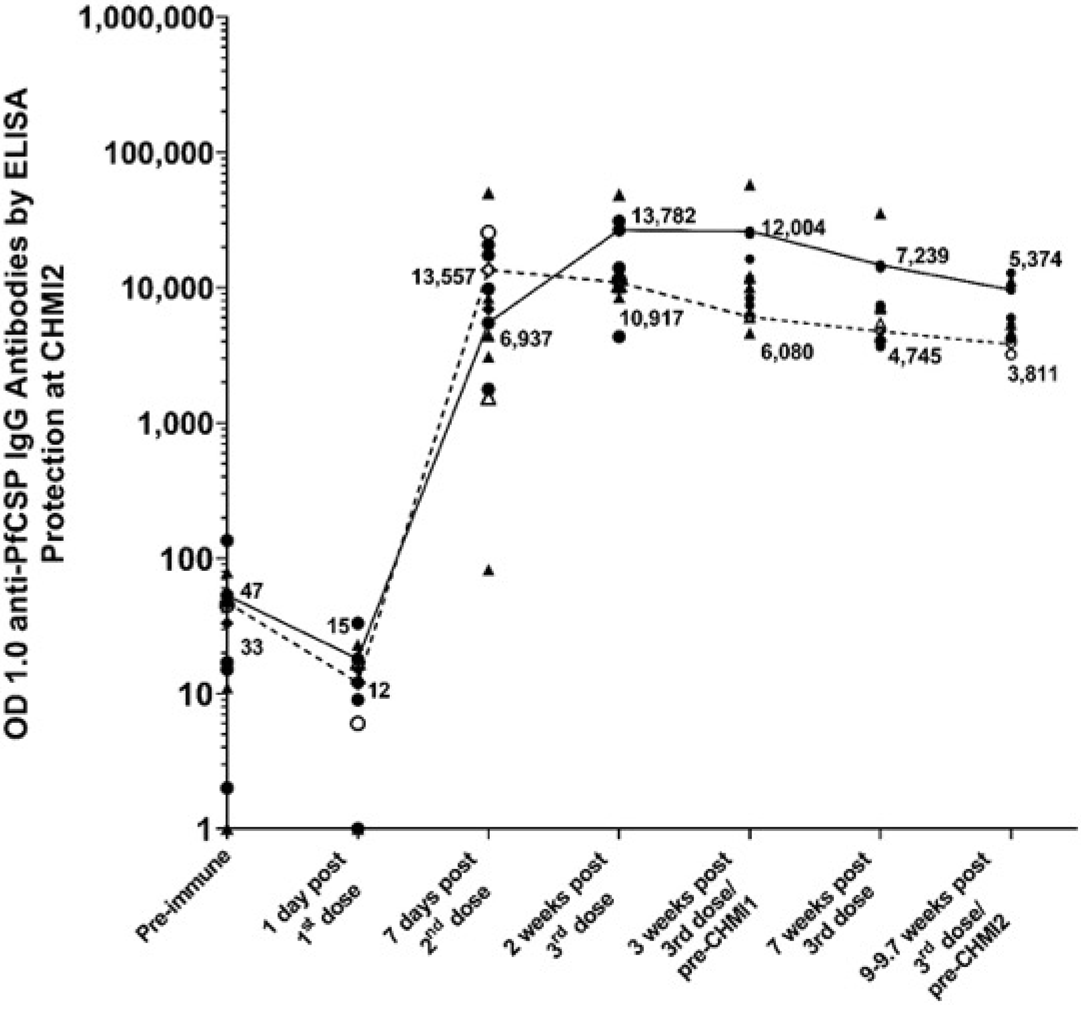


**Supplementary Figure 2.** ***Verification phase antibody kinetics***: OD 1.0 for IgG antibodies to PfCSP measured at different intervals post PfSPZ Vaccine. Filled triangles are uninfected subjects and open triangles are infected subjects who received heterologous CHMI with PfSPZ Challenge (7G8) and filled circles are uninfected subjects and open circles are infected subjects who received homologous CHMI with PfSPZ Challenge (NF54). Solid line is conncting the median OD 1.0 values for all protected subjects and dotted line is connecting median OD 1.0 values for uninfected subjects.

**Supplementary Table 1. Number and Percentage of Participants Experiencing Solicited Adverse Events During the Optimization Phase.** All solicited AE are treated as related.

| **All doses** | | **Group A (n = 6)** | | **Group B1 (n = 6)** | | **Group C2 (n= 6)** | |
|---|---|---|---|---|---|---|---|
| **Solicited Adverse Event** | **Severity** | **n** | **%** | **n** | **%** | **n** | **%** |
| **Any Adverse Event** | Mild | 4 | 66.7 | 0 | 0 | 1 | 16.7 |
| | Moderate | 1 | 16.7 | 4 | 66.7 | 2 | 33.3 |
| | Severe | 1 | 16.7 | 0 | 0 | 1 | 16.7 |
| **Any Systemic Adverse Event** | Mild | 4 | 66.7 | 0 | 0 | 1 | 16.7 |
| | Moderate | 0 | 0 | 4 | 66.7 | 1 | 16.7 |
| | Severe | 1 | 16.7 | 0 | 0 | 1 | 16.7 |
| Nausea | Mild | 1 | 16.7 | 0 | 0 | 0 | 0 |
| | Moderate | 0 | 0 | 0 | 0 | 0 | 0 |
| | Severe | 0 | 0 | 0 | 0 | 0 | 0 |
| Diarrhea | Mild | 0 | 0 | 1 | 16.7 | 0 | 0 |
| | Moderate | 0 | 0 | 0 | 0 | 0 | 0 |
| | Severe | 0 | 0 | 0 | 0 | 0 | 0 |
| Fatigue | Mild | 4 | 66.7 | 3 | 50 | 2 | 33.3 |
| | Moderate | 0 | 0 | 0 | 0 | 0 | 0 |
| | Severe | 0 | 0 | 0 | 0 | 1 | 16.7 |
| Headache | Mild | 0 | 0 | 0 | 0 | 1 | 16.7 |
| | Moderate | 4 | 66.7 | 3 | 50 | 1 | 16.7 |
| | Severe | 0 | 0 | 0 | 0 | 0 | 0 |
| Myalgia | Mild | 1 | 16.7 | 2 | 33.3 | 0 | 0 |
| | Moderate | 0 | 0 | 0 | 0 | 0 | 0 |
| | Severe | 0 | 0 | 0 | 0 | 1 | 16.7 |
| Fever (including Subjective Fever) | Mild | 0 | 0 | 3 | 50 | 1 | 16.7 |
| | Moderate | 0 | 0 | 0 | 0 | 0 | 0 |
| | Severe | 1 | 16.7 | 0 | 0 | 0 | 0 |
| Malaise | Mild | 0 | 0 | 0 | 0 | 0 | 0 |
| | Moderate | 0 | 0 | 0 | 0 | 0 | 0 |
| | Severe | 0 | 0 | 0 | 0 | 0 | 0 |
| Chills | Mild | 2 | 33.3 | 1 | 16.7 | 0 | 0 |
| | Moderate | 0 | 0 | 0 | 0 | 1 | 16.7 |
| | Severe | 0 | 0 | 0 | 0 | 1 | 16.7 |
| Sweats | Mild | 0 | 0 | 2 | 33.3 | 0 | 0 |
| | Moderate | 0 | 0 | 0 | 0 | 0 | 0 |
| | Severe | 0 | 0 | 0 | 0 | 0 | 0 |
| Arthralgia | Mild | 1 | 16.7 | 1 | 16.7 | 0 | 0 |
| | Moderate | 1 | 16.7 | 0 | 0 | 0 | 0 |
| | Severe | 0 | 0 | 0 | 0 | 0 | 0 |
| Vomiting | Mild | 0 | 0 | 0 | 0 | 0 | 0 |
| | Moderate | 0 | 0 | 0 | 0 | 0 | 0 |
| | Severe | 0 | 0 | 0 | 0 | 1 | 16.7 |
| Dizziness | Mild | 3 | 50 | 0 | 0 | 0 | 0 |
| | Moderate | 0 | 0 | 0 | 0 | 0 | 0 |
| | Severe | 0 | 0 | 0 | 0 | 0 | 0 |
| **Any Local Adverse Event** | Mild | 2 | 33.3 | 0 | 0 | 2 | 33.3 |
| | Moderate | 0 | 0 | 0 | 0 | 1 | 16.7 |
| | Severe | 0 | 0 | 0 | 0 | 0 | 0 |
| Bruising | Mild | 0 | 0 | 0 | 0 | 1 | 16.7 |
| | Moderate | 0 | 0 | 0 | 0 | 0 | 0 |
| | Severe | 0 | 0 | 0 | 0 | 0 | 0 |
| Hematoma | Mild | 0 | 0 | 0 | 0 | 0 | 0 |
| | Moderate | 0 | 0 | 0 | 0 | 0 | 0 |
| | Severe | 0 | 0 | 0 | 0 | 0 | 0 |
| Pain | Mild | 1 | 16.7 | 0 | 0 | 1 | 16.7 |

| | | | | | | | |
|---|---|---|---|---|---|---|---|
| | Moderate | 0 | 0 | 0 | 0 | 1 | 16.7 |
| | Severe | 0 | 0 | 0 | 0 | 0 | 0 |
| Pruritus | Mild | 1 | 16.7 | 0 | 0 | 0 | 0 |
| | Moderate | 0 | 0 | 0 | 0 | 0 | 0 |
| | Severe | 0 | 0 | 0 | 0 | 0 | 0 |

**Supplementary Table 2. Number and Percentage of Participants Experiencing Unsolicited Adverse Events Considered Possibly, Probably, or Definitely Related by MedDRA® System Organ Class, Severity, and Treatment Group – through 21 Days After the 3rd Immunization (Labs and Vital Signs Excluded).**

| All doses | | Group A (n = 6) | | Group B1 (n = 6) | | Group C2 (n= 6) | |
|---|---|---|---|---|---|---|---|
| **MedDRA® System Organ Class** | **Severity** | **n** | **%** | **n** | **%** | **n** | **%** |
| Any SOC | None | 0 | 0 | 4 | 66.7 | 5 | 83.4 |
| | Mild | 5 | 83.4 | 2 | 33.3 | 1 | 16.6 |
| | Moderate | 1 | 16.6 | 0 | 0 | 0 | 0 |
| | Severe | 0 | 0 | 0 | 0 | 0 | 0 |
| Gastrointestinal disorders | None | 5 | 83.4 | 5 | 83.4 | 6 | 100 |
| | Mild | 0 | 0 | 1 | 16.6 | 0 | 0 |
| | Moderate | 1 | 16.6 | 0 | 0 | 0 | 0 |
| | Severe | 0 | 0 | 0 | 0 | 0 | 0 |
| Blood and lymphatic system disorders | None | 5 | 83.4 | 6 | 100 | 6 | 100 |
| | Mild | 1 | 16.6 | 0 | 0 | 0 | 0 |
| | Moderate | 0 | 0 | 0 | 0 | 0 | 0 |
| | Severe | 0 | 0 | 0 | 0 | 0 | 0 |
| Musculoskeletal and connective tissue disorders | None | 5 | 83.4 | 6 | 100 | 6 | 100 |
| | Mild | 1 | 16.6 | 0 | 0 | 0 | 0 |
| | Moderate | 0 | 0 | 0 | 0 | 0 | 0 |
| | Severe | 0 | 0 | 0 | 0 | 0 | 0 |
| Nervous system disorders | None | 4 | 66.7 | 6 | 100 | 6 | 100 |
| | Mild | 2 | 33.3 | 0 | 0 | 0 | 0 |
| | Moderate | 0 | 0 | 0 | 0 | 0 | 0 |
| | Severe | 0 | 0 | 0 | 0 | 0 | 0 |
| General disorders and administration site conditions | None | 6 | 100 | 5 | 83.4 | 6 | 100 |
| | Mild | 0 | 0 | 1 | 16.6 | 0 | 0 |
| | Moderate | 0 | 0 | 0 | 0 | 0 | 0 |
| | Severe | 0 | 0 | 0 | 0 | 0 | 0 |
| Infections and infestations | None | 2 | 33.3 | 5 | 83.4 | 6 | 100 |
| | Mild | 4 | 66.7 | 1 | 16.6 | 0 | 0 |
| | Moderate | 0 | 0 | 0 | 0 | 0 | 0 |
| | Severe | 0 | 0 | 0 | 0 | 0 | 0 |
| Respiratory, thoracic and mediastinal disorders | None | 5 | 83.4 | 6 | 100 | 6 | 100 |
| | Mild | 1 | 16.6 | 0 | 0 | 0 | 0 |
| | Moderate | 0 | 0 | 0 | 0 | 0 | 0 |
| | Severe | 0 | 0 | 0 | 0 | 0 | 0 |
| Psychiatric disorders | None | 5 | 83.4 | 6 | 100 | 6 | 100 |
| | Mild | 1 | 16.6 | 0 | 0 | 0 | 0 |

| All doses | | Group A (n = 6) | | Group B1 (n = 6) | | Group C2 (n= 6) | |
|---|---|---|---|---|---|---|---|
| **MedDRA® System Organ Class** | **Severity** | **n** | **%** | **n** | **%** | **n** | **%** |
| | Moderate | 0 | 0 | 0 | 0 | 0 | 0 |
| | Severe | 0 | 0 | 0 | 0 | 0 | 0 |
| Cardiac disorders | None | 4 | 66.7 | 5 | 83.4 | 6 | 100 |
| | Mild | 2 | 33.3 | 1 | 16.6 | 0 | 0 |
| | Moderate | 0 | 0 | 0 | 0 | 0 | 0 |
| | Severe | 0 | 0 | 0 | 0 | 0 | 0 |
| Skin and subcutaneous tissue disorders | None | 5 | 83.4 | 6 | 100 | 6 | 100 |
| | Mild | 1 | 16.6 | 0 | 0 | 0 | 0 |
| | Moderate | 0 | 0 | 0 | 0 | 0 | 0 |
| | Severe | 0 | 0 | 0 | 0 | 0 | 0 |
| Immune system disorders | None | 6 | 100 | 6 | 100 | 6 | 100 |
| | Mild | 0 | 0 | 0 | 0 | 0 | 0 |
| | Moderate | 0 | 0 | 0 | 0 | 0 | 0 |
| | Severe | 0 | 0 | 0 | 0 | 0 | 0 |
| Injury, poisoning and procedural complications | None | 6 | 100 | 6 | 100 | 5 | 83.4 |
| | Mild | 0 | 0 | 0 | 0 | 1 | 16.6 |
| | Moderate | 0 | 0 | 0 | 0 | 0 | 0 |
| | Severe | 0 | 0 | 0 | 0 | 0 | 0 |

**Supplementary Table 3. Number and Percentage of Participants with Abnormal Hematology or Biochemistry Laboratory Results through 21 Days After the Third Immunization- *Optimization* and *Verification* Phases.**

| | | Optimization (n = 18) | | Verification | | | |
|---|---|---|---|---|---|---|---|
| | | | | PfSPZ (n = 12) | | Placebo (n = 6) | |
| **Lab parameter** | **Severity** | **n** | **%** | **n** | **%** | **n** | **%** |
| Hemoglobin, decreased | Mild | 5 | 28 | 6 | 50 | 4 | 67 |
| | Moderate | 0 | 0 | 0 | 0 | 0 | 0 |
| | Severe | 0 | 0 | 0 | 0 | 0 | 0 |
| Leucocytes, decreased | Mild | 3 | 17 | 1 | 8.3 | 0 | 0 |
| | Moderate | 1 | 5.6 | 0 | 0 | 0 | 0 |
| | Severe | 0 | 0 | 0 | 0 | 0 | 0 |
| Platelets, decreased | Mild | 1 | 5.6 | 1 | 8.3 | 0 | 0 |
| | Moderate | 0 | 0 | 0 | 0 | 0 | 0 |
| | Severe | 0 | 0 | 0 | 0 | 0 | 0 |
| Neutrophils, decreased | Mild | 3 | 17 | 4 | 33 | 0 | 0 |
| | Moderate | 1 | 5.6 | 1 | 8.3 | 1 | 17 |
| | Severe | 0 | 0 | 0 | 0 | 0 | 0 |
| Eosinophils, increased | Mild | 0 | 0 | 0 | 0 | 0 | 0 |
| | Moderate | 0 | 0 | 0 | 0 | 0 | 0 |
| | Severe | 0 | 0 | 0 | 0 | 0 | 0 |
| Lymphocytes, decreased[1] | Mild | 5 | 28 | 3 | 25 | 0 | 0 |
| | Moderate | 3 | 17 | 7 | 58 | 0 | 0 |
| | Severe | 2 | 11 | 0 | 0 | 0 | 0 |
| Total Bilirubin, increased | Mild | 0 | 0 | 1 | 8 | 0 | 0 |
| | Moderate | 0 | 0 | 0 | 0 | 0 | 0 |
| | Severe | 0 | 0 | 0 | 0 | 0 | 0 |
| Creatinine, increased | Mild | 0 | 0 | 0 | 0 | 0 | 0 |
| | Moderate | 0 | 0 | 0 | 0 | 0 | 0 |
| | Severe | 0 | 0 | 0 | 0 | 0 | 0 |
| AST (SGOT), increased | Mild | 1 | 5.6 | 2 | 16 | 0 | 0 |
| | Moderate | 0 | 0 | 0 | 0 | 0 | 0 |
| | Severe | 0 | 0 | 0 | 0 | 0 | 0 |
| ALT (SGPT), increased | Mild | 1 | 5.6 | 3 | 25 | 0 | 0 |
| | Moderate | 0 | 0 | 0 | 0 | 0 | 0 |
| | Severe | 0 | 0 | 0 | 0 | 0 | 0 |
| Glucose, increased | Mild | 1 | 5.5 | 0 | 0 | 0 | 0 |
| | Moderate | 1 | 5.5 | 1 | 16 | 0 | 0 |
| | Severe | 0 | 0 | 0 | 0 | 0 | 0 |

| | | Optimization (n = 18) | | Verification | | | |
|---|---|---|---|---|---|---|---|
| | | | | PfSPZ (n = 12) | | Placebo (n = 6) | |
| **Lab parameter** | **Severity** | **n** | **%** | **n** | **%** | **n** | **%** |
| Glucose, decreased | Mild | 2 | 11 | 1 | 8.3 | 1 | 16 |
| | Moderate | 2 | 11 | 5 | 41 | 2 | 32 |
| | Severe | 1[2] | 5.5 | 0 | 0 | 0 | 0 |
| Sodium, increased | Mild | 2 | 11 | 0 | 0 | 0 | 0 |
| | Moderate | 0 | 0 | 0 | 0 | 0 | 0 |
| | Severe | 0 | 0 | 0 | 0 | 0 | 0 |
| Sodium, decreased | Mild | 0 | 0 | 0 | 0 | 0 | 0 |
| | Moderate | 0 | 0 | 0 | 0 | 0 | 0 |
| | Severe | 0 | 0 | 0 | 0 | 0 | 0 |
| Potassium, increased | Mild | 1 | 5.5 | 0 | 0 | 0 | 0 |
| | Moderate | 0 | 0 | 0 | 0 | 0 | 0 |
| | Severe | 0 | 0 | 0 | 0 | 0 | 0 |
| Potassium, decreased | Mild | 0 | 0 | 0 | 0 | 0 | 0 |
| | Moderate | 0 | 0 | 0 | 0 | 0 | 0 |
| | Severe | 0 | 0 | 1[3] | 8.3 | 0 | 0 |

[1]Verification phase, PfSPZ Vaccine vs. NS, p=0.015 Fisher's exact test, 2 tailed. 19/20 abnormal lymphocyte counts occurred 1 day after a vaccine dose and had returned towards normal at the next measurement.
[2]Asymptomatic hypoglycemia with glucose 48 mg/dL, resolved.
[3]Hypokalemia (3.2 mmol/L) 20 days after the 3rd dose, resolved.

**Supplementary Table 4. Related Moderate (Grade 2) and Severe (Grade 3) Adverse Events, Vital Sign and Laboratory Abnormalities Beginning on Day 6 Following Inoculation of PfSPZ Challenge for CHMI by Cohort and Absence of Parasitaemia (Negative) or Presence of Parasitaemia According to Pf (PfNF54 or Pf7G8) used for CHMI.** For the vaccine and placebo groups the results from CHMI 1 and CHMI 2 are combined.

| Preferred term | Severity grade | Optimization (17 underwent CHMI with NF54) | | Verification: Vaccine (12 underwent CHMI twice, 24 CHMIs) | | | Verification: Placebo (6 underwent CHMI twice, 12 CHMIs) | | |
|---|---|---|---|---|---|---|---|---|---|
| | | Negative (N=12) | NF54 (N=5) | Negative (N=19) | 7G8 (N=2) | NF54 (N=3) | Negative (N=1) | 7G8 (N=6) | NF54 (N=5) |
| Chills | Moderate | 0 | 0 | 0 | 0 | 0 | 0 | 0 | 1 |
| | Severe | 0 | 0 | 0 | 0 | 0 | 0 | 0 | 0 |
| Headache | Moderate | 0 | 0 | 2 | 0 | 1 | 1 | 2 | 2 |
| | Severe | 0 | 0 | 0 | 0 | 0 | 0 | 0 | 0 |
| Myalgia | Moderate | 0 | 0 | 0 | 0 | 0 | 0 | 1 | 1 |
| | Severe | 0 | 0 | 0 | 0 | 0 | 0 | 0 | 0 |
| Nausea | Moderate | 0 | 0 | 0 | 0 | 0 | 0 | 1 | 0 |
| | Severe | 0 | 0 | 0 | 0 | 0 | 0 | 0 | 0 |
| Pyrexia** | Moderate | 0 | 0 | 0 | 1 | 0 | 0 | 1 | 2 |
| | Severe | 0 | 0 | 0 | 0 | 0 | 0 | 0 | 0 |
| Abdominal pain | Moderate | 1 | 0 | 0 | 0 | 0 | 0 | 0 | 0 |
| | Severe | 0 | 0 | 0 | 0 | 0 | 0 | 0 | 0 |
| Tachycardia | Moderate | 1 | 0 | 0 | 0 | 0 | 0 | 0 | 0 |
| | Severe | 0 | 0 | 0 | 0 | 0 | 0 | 0 | 0 |
| Leukopenia | Moderate | 0 | 0 | 0 | 0 | 0 | 0 | 1 | 0 |
| | Severe | 0 | 1 | 0 | 0 | 0 | 0 | 0 | 0 |
| Lymphopenia | Moderate | 0 | 0 | 0 | 0 | 1 | 0 | 0 | 1 |
| | Severe | 0 | 1 | 0 | 0 | 0 | 0 | 2 | 1 |
| Neutropenia | Moderate | 0 | 0 | 0 | 0 | 1 | 0 | 0 | 0 |
| | Severe | 0 | 0 | 0 | 0 | 0 | 0 | 1 | 0 |

*There were two severe (Grade 3) (lymphopenia and leukopenia) AEs during *Optimization* that occurred in subjects who had parasitemia and one severe (Grade 3) AE (lymphopenia) during *Verification* that occurred in a subject who had parasitemia – all of which resolved.
**Includes subjective report of fever and/or a measured abnormal temperature.

**Supplementary Table 5. Prepatent Periods and Parasite Density by qPCR in Participants Infected in the Verfication Phase, CHMI 1 and 2.**

| Subject | PfSPZ or NS | CHMI 1 | | | CHMI 2 | | |
|---|---|---|---|---|---|---|---|
| | | **Strain** | **Prepatent Period - days** | **Parasite Density – Pf/mL** | **Strain** | **Prepatent Period - days** | **Parasite Density – Pf/mL** |
| 056 | PfSPZ | 7G8 | 10 | 181 | NF54 | Not infected | |
| 059 | PfSPZ | NF54 | 11 | 2503 | 7G8 | 10 | 371 |
| 060 | PfSPZ | NF54 | 16 | 681 | 7G8 | Not infected | |
| 076 | PfSPZ | 7G8 | Not infected | | NF54 | 10 | 505 |
| 054 | NS | NF54 | 9 | 216 | 7G8 | 9 | 781 |
| 058 | NS | 7G8 | 9 | 970 | NF54 | Not infected | |
| 065 | NS | NF54 | 9 | 274 | 7G8 | 9 | 342 |
| 067 | NS | 7G8 | 9 | 454 | NF54 | 13 | 1524 |
| 073 | NS | 7G8 | 9 | 163 | NF54 | 13 | 1235 |
| 075 | NS | NF54 | 9 | 477 | 7G8 | 11 | 2669 |

**Supplementary Table 6. Immunological data for all volunteers from the optimization phase of the MAVACHE clinical trial, as measured by PfCSP ELISA assay.**

All out-of-range values, negatives and zeroes are reported as 1.

| Group | | PfSPZ/Dose | ID | CHMI | Infected | ELISA | | | | | | | | | |
|---|---|---|---|---|---|---|---|---|---|---|---|---|---|---|---|
| | | | | | | PfCSP OD 1.0 | | | | | | | | | |
| | | | | | | Pre-Immune | 2 weeks post 3$^{rd}$ dose | Net (Post-Pre) | Ratio (Post/Pre) | Pre-CHMI 1 | Pre-CHMI 1 Net | Pre-CHMI 1 Ratio | Pre-CHMI 2 | Pre-CHMI 2 Net | Pre-CHMI 2 Ratio |
| Optimization | A | Group A: 9.0 X $10^5$ PfSPZ in 1.0 mL administered DVI at Day 0, 7, and 28. | M1.002 | NF54 | No | 76 | 29,309 | 29,233 | 385 | 14,121 | 14,045 | 185 | | | |
| | | | M1.003 | NF54 | No | 26 | 31,640 | 31,614 | 1,216 | 19,191 | 19,165 | 737 | | | |
| | | | M1.004 | NF54 | No | 55 | 11,834 | 11,779 | 214 | 24,465 | 24,410 | 444 | | | |
| | | | M1.006 | NF54 | No | 50 | 17,964 | 17,914 | 358 | 13,734 | 13,684 | 274 | | | |
| | | | M1.009 | NF54 | No | 26 | 78,430 | 78,404 | 3,016 | 68,269 | 68,243 | 2,625 | | | |
| | B1 | Group B1: 1.35 X $10^6$ PfSPZ in 1.0 mL administered DVI at Day 0, and 7. | M1.014 | NF54 | No | 57 | 16,649 | 16,592 | 291 | 297 | 240 | 4 | | | |
| | | | M1.018 | NF54 | Yes | 125 | 9,982 | 9,857 | 79 | 12,820 | 12,695 | 102 | | | |
| | | | M1.026 | NF54 | No | 92 | 9,760 | 9,668 | 105 | 13,067 | 12,975 | 141 | | | |
| | | | M1.027 | NF54 | No | 31 | 9,453 | 9,422 | 304 | 5,368 | 5,337 | 172 | | N/A | |
| | | | M1.032 | NF54 | Yes | 71 | 4,465 | 4,394 | 62 | 8,207 | 8,136 | 115 | | | |
| | | | M1.033 | NF54 | No | 33 | 19,518 | 19,485 | 590 | 4,189 | 4,156 | 126 | | | |
| | C2 | Group C2: 2.7 X $10^6$ PfSPZ in 1.0 mL administered DVI at Day 0, and 7. | M1.039 | NF54 | No | 1 | 27,561 | 27,560 | 27,560 | 19,685 | 19,684 | 19,684 | | | |
| | | | M1.041 | NF54 | Yes | 44 | 10,110 | 10,066 | 229 | 7,708 | 7,664 | 174 | | | |
| | | | M1.044 | NF54 | Yes | 49 | 4,930 | 4,881 | 100 | 10,320 | 10,271 | 210 | | | |
| | | | M1.046 | NF54 | No | 56 | 44,275 | 44,219 | 790 | 14,283 | 14,227 | 254 | | | |
| | | | M1.047 | NF54 | No | 1,101 | 49,338 | 48,237 | 44 | 40,667 | 39,566 | 36 | | | |
| | | | M1.048 | NF54 | Yes | 1 | 2,280 | 2,279 | 2,279 | 2,891 | 2,890 | 2,890 | | | |

**Supplementary Table 7. Increased levels as compared to pre-immunization for IgG antibodies to PfCSP by ELISA, IgG antibodies to PfSPZ by aIFA and inhibition of PfSPZ invasion of hepatocytes by aISI in sera taken one day prior to the first CHMI 3 weeks after the last dose of vaccine and one day prior to the second CHMI 9-10 weeks after the last dose of vaccine**. For ELISA, samples were considered to have increased (seroconverted) over pre-immunization, if the difference between the post-immunization OD 1.0 and the pre-immunization OD 1.0 (net OD 1.0) was ≥50 and the ratio of the post-immunization OD 1.0 to pre-immunization OD 1.0 (ratio) was ≥3.0. For automated immunofluorescence assays, participants with a net reciprocal serum dilution for $2.0x10^5$ arbitrary fluorescence units (AFU) of ≥150 and a ratio reciprocal serum dilution of ≥3.0 were considered positive. For the automated inhibition of sporozoite invasion assay, participants with a net reciprocal serum dilution for 80% inhibition of ≥10 in the inhibition of sporozoite invasion assay and a ratio reciprocal serum dilution for 80% inhibition of ≥3.0 in the inhibition of sporozoite invasion assay were considered positive.

| | **PfCSP ELISA** | | **PfSPZ aIFA** | | **PfSPZ aISI** | |
|---|---|---|---|---|---|---|
| | **CHMI 1** | **CHMI 2** | **CHMI 1** | **CHMI 2** | **CHMI 1** | **CHMI 2** |
| **Uninfected (no. with significant increase/no. vaccinated)** | 9/9 (100%) | 9/9 (100%) | 6/9 (66.7%) | 8/9 (88.9%) | 9/9 (100%) | 9/9 (100%) |
| **Infected (no. with significant increase/no. vaccinated)** | 3/3 (100%) | 3/3 (100%) | 3/3 (100%) | 3/3 (100%) | 3/3 (100%) | 3/3 (100%) |

**Supplementary Table 8. Immunological data for all volunteers who received three doses of $9.0x10^5$ PfSPZ Vaccine or saline in the verification phase of MAVACHE clinical trial, as measured by PfCSP ELISA, aIFA, and ISI assays.**

All out-of-range values, negatives and zeroes are reported as 1. Three subjects (shown in italics below): [1]no sample available 2 weeks post 3rd dose (n=1) sample included in the analysis was drawn 1 day post 3rd dose for IgG and IgM ELISA, [2]no sample available 2 weeks post 3rd dose (n=2).

| Group | PfSPZ/Dose | ID | CHMI | Infected | ELISA | | | | | | | | | |
|---|---|---|---|---|---|---|---|---|---|---|---|---|---|---|
| | | | | | PfCSP OD 1.0 | | | | | | | | | |
| | | | | | Pre-Immune | 2 weeks post 3rd dose | Net (Post-Pre) | Ratio (Post/Pre) | Pre-CHMI 1 | Pre-CHMI 1 Net | Pre-CHMI 1 Ratio | Pre-CHMI 2 | Pre-CHMI 2 Net | Pre-CHMI 2 Ratio |
| Verification | $9.0 X 10^5$ PfSPZ in 1.0 mL administered DVI at Day 0, 7, and 28. | M1.051 | NF54/7G8 | No/No | 58 | 10,025 | 9,967 | 173 | 12,174 | 12,116 | 210 | 5,405 | 5,347 | 93 |
| | | M1.060 | NF54/7G8 | Yes/No | 11 | 8,526 | 8,515 | 775 | 4,618 | 4,607 | 420 | 4,661 | 4,650 | 424 |
| | | M1.063 | NF54/7G8 | No/No | 79 | 13,590 | 13,511 | 172 | 10,083 | 10,004 | 128 | 5,342 | 5,263 | 68 |
| | | M1.070 | NF54/7G8 | No/No | 49 | 48,674 | 48,625 | 993 | 57,880 | 57,831 | 1,181.00 | 11,274 | 11,225 | 230 |
| | | M1.071 | NF54/7G8 | No/No | 1 | 12,203 | 12,202 | 12,203.00 | 11,834 | 11,833 | 11,834.00 | 4,739 | 4,738 | 4,739.00 |
| | | M1.056 | 7G8/NF54 | Yes/No | 2 | 14,042 | 14,040 | 7,021.00 | 16,248 | 16,246 | 8,124.00 | 4,314 | 4,312 | 2,157.00 |
| | | M1.049 | 7G8/NF54 | No/No | 135 | 13,973 | 13,838 | 104 | 8,353 | 8,218 | 62 | 5,978 | 5,843 | 44 |
| | | M1.050 | 7G8/NF54 | No/No | 17 | 4,345 | 4,328 | 256 | 7,398 | 7,381 | 435 | 4,202 | 4,185 | 247 |
| | | M1.064 | 7G8/NF54 | No/No | 15 | *341,171* | 34,102 | 2,274.50 | 24,893 | 24,878 | 1,660.00 | 12,836 | 12,821 | 856 |
| | | M1.086 | 7G8/NF54 | No/No | 53 | 26,632 | 26,579 | 502 | 26,227 | 26,174 | 495 | 9,642 | 9,589 | 182 |
| | | M1.076 | 7G8/NF54 | No/Yes | 45 | 11,359 | 11,314 | 252 | 6,134 | 6,089 | 136 | 3,204 | 3,159 | 71 |
| | | M1.059 | NF54/7G8 | Yes/Yes | 49 | 10,475 | 10,426 | 214 | 6,786 | 6,737 | 138.5 | 4,418 | 4,369 | 90 |
| | Placebo | M1.054 | NF54/7G8 | Yes/Yes | 60 | 97 | 37 | 2 | 64 | 4 | 1 | 168 | 108 | 3 |
| | | M1.065 | NF54/7G8 | Yes/No | 8 | *11* | 1 | 1 | 1 | 1 | 1 | 1 | 1 | 1 |
| | | M1.075 | NF54/7G8 | Yes/Yes | 18 | 13 | 1 | 1 | 1 | 1 | 1 | 64 | 46 | 4 |
| | | M1.058 | 7G8/NF54 | Yes/Yes | 44 | 43 | 1 | 1 | 31 | 1 | 1 | 65 | 21 | 1 |
| | | M1.067 | 7G8/NF54 | Yes/Yes | 19 | 1 | 1 | 1 | 23 | 4 | 1 | 80 | 61 | 4 |
| | | M1.073 | 7G8/NF54 | Yes/Yes | 98 | *631* | 1 | 1 | 72 | 1 | 1 | 180 | 82 | 2 |

| Group | PfSPZ/Dose | ID | CHMI | Infected | ELISA | | | | | | | | | |
|---|---|---|---|---|---|---|---|---|---|---|---|---|---|---|
| | | | | | PfCSP IgM OD 1.0 | | | | | | | | | |
| | | | | | Pre-Immune | 2 weeks post 3rd dose | Net (Post-Pre) | Ratio (Post/Pre) | Pre-CHMI 1 | Pre-CHMI 1 Net | Pre-CHMI 1 Ratio | Pre-CHMI 2 | Pre-CHMI 2 Net | Pre-CHMI 2 Ratio |
| Verification | **9.0 X $10^5$ PfSPZ in 1.0 mL administered DVI at Day 0, 7, and 28.** | M1.051 | NF54/7G8 | No/No | 127 | 234,942 | 234,815 | 1,850.00 | 273,707 | 273,580 | 2,155.00 | 38,222 | 38,095 | 301 |
| | | M1.060 | NF54/7G8 | Yes/No | 275 | 181,989 | 181,714 | 662 | 191,043 | 190,768 | 695 | 46,326 | 46,051 | 168 |
| | | M1.063 | NF54/7G8 | No/No | 391 | 445,622 | 445,231 | 1,140.00 | 124,433 | 124,042 | 318 | 53,185 | 52,794 | 136 |
| | | M1.070 | NF54/7G8 | No/No | 940 | 825,681 | 824,741 | 878 | 285,116 | 284,176 | 303 | 172,993 | 172,053 | 184 |
| | | M1.071 | NF54/7G8 | No/No | 144 | 1,063,618 | 1,063,474 | 7,386.00 | 1,556,905 | 1,556,904 | 10,812.00 | 62,729 | 62,728 | 436 |
| | | M1.056 | 7G8/NF54 | Yes/No | 512 | 332,879 | 332,367 | 650 | 332,879 | 800,863 | 1,565.00 | 332,879 | 89,908 | 177 |
| | | M1.049 | 7G8/NF54 | No/No | 1,715 | 454,731 | 453,016 | 265 | 148,375 | 146,660 | 87 | 106,631 | 104,916 | 62 |
| | | M1.050 | 7G8/NF54 | No/No | 69 | 49,690 | 49,621 | 720 | 55,708 | 55,639 | 807 | 23,132 | 23,063 | 335 |
| | | M1.064 | 7G8/NF54 | No/No | 157 | *2,661,961* | 266,039 | 1,695.50 | 159,112 | 158,955 | 1,013.00 | 40,788 | 40,631 | 260 |
| | | M1.086 | 7G8/NF54 | No/No | 592 | 558,654 | 558,062 | 944 | 195,990 | 195,398 | 331 | 92,709 | 92,117 | 157 |
| | | M1.076 | 7G8/NF54 | No/Yes | 1 | 259,625 | 259,624 | 259,625.00 | 273,757 | 273,756 | 273,757.00 | 32,240 | 32,239 | 32,240.00 |
| | | M1.059 | NF54/7G8 | Yes/Yes | 388 | 168,220 | 167,832 | 434 | 109,861 | 109,473 | 283 | 51,011 | 50,623 | 131 |
| | **Placebo** | M1.054 | NF54/7G8 | Yes/Yes | 159 | 134 | 1 | 1 | 140 | 1 | 1 | 373 | 214 | 2 |
| | | M1.065 | NF54/7G8 | Yes/No | 149 | *11* | 1 | 1 | 162 | 1 | 1 | 158 | 19 | 1 |
| | | M1.075 | NF54/7G8 | Yes/Yes | 18 | 13 | 1 | 1 | 1 | 1 | 1 | 64 | 46 | 4 |
| | | M1.058 | 7G8/NF54 | Yes/Yes | 208 | 215 | 7 | 1 | 202 | 1 | 1 | 570 | 362 | 3 |
| | | M1.067 | 7G8/NF54 | Yes/Yes | 123 | 229 | 1 | 2 | 201 | 78 | 2 | 1,259 | 1,136 | 10 |
| | | M1.073 | 7G8/NF54 | Yes/Yes | 477 | *321* | 1 | 1 | 960 | 483 | 2 | 7,858 | 7,381 | 16 |

| Group | PfSPZ/Dose | ID | CHMI | Infected | aIFA | | | | | | | | | |
|---|---|---|---|---|---|---|---|---|---|---|---|---|---|---|
| | | | | | AFU 2x$10^5$ | | | | | | | | | |
| | | | | | Pre-Immune | 2 weeks post 3rd dose | Net (Post-Pre) | Ratio (Post/Pre) | Pre-CHMI 1 | Pre-CHMI 1 Net | Pre-CHMI 1 Ratio | Pre-CHMI 2 | Pre-CHMI 2 Net | Pre-CHMI 2 Ratio |
| Verification | 9.0 X $10^5$ PfSPZ in 1.0 mL administered DVI at Day 0, 7, and 28. | M1.051 | NF54/7G8 | No/No | 1 | 12,194 | 12,193 | 12,194.00 | 18,730 | 18,729 | 18,730.00 | 13,009 | 13,008 | 13,009.00 |
| | | M1.060 | NF54/7G8 | Yes/No | 1 | 8,410 | 8,409 | 8,410.00 | 7,704 | 7,703 | 7,704.00 | 6,213 | 6,212 | 6,213.00 |
| | | M1.063 | NF54/7G8 | No/No | 205 | 405 | 200 | 2 | 147 | -59 | 1 | 76,256 | 76,050 | 371 |
| | | M1.070 | NF54/7G8 | No/No | 375 | 863 | 488 | 2 | 228 | -147 | 1 | 1 | 1 | 1 |
| | | M1.071 | NF54/7G8 | No/No | 170 | 28,284 | 28,114 | 167 | 26,261 | 26,091 | 155 | 10,513 | 10,343 | 62 |
| | | M1.056 | 7G8/NF54 | Yes/No | 43 | 23,869 | 23,826 | 560 | 18,000 | 17,957 | 422 | 8,359 | 8,317 | 196 |
| | | M1.049 | 7G8/NF54 | No/No | 33 | 31,107 | 31,074 | 929 | 12,847 | 12,814 | 384 | 7,123 | 7,089 | 213 |
| | | M1.050 | 7G8/NF54 | No/No | 50 | 6,369 | 6,319 | 127 | 8,705 | 8,655 | 174 | 2,637 | 2,586 | 53 |
| | | M1.064 | 7G8/NF54 | No/No | 80 | *NS*[2] | *NS*[2] | *NS*[2] | 19,132 | 19,052 | 239 | 13,875 | 13,795 | 174 |
| | | M1.086 | 7G8/NF54 | No/No | 67 | 34,072 | 34,005 | 509 | 1 | -66 | 1 | 13,838 | 13,771 | 207 |
| | | M1.076 | 7G8/NF54 | No/Yes | 177 | 25,603 | 25,427 | 145 | 23,324 | 23,148 | 132 | 4,032 | 3,855 | 23 |
| | | M1.059 | NF54/7G8 | Yes/Yes | 43 | 23,869 | 15,144 | 15,145.00 | 18,000 | 12,539 | 12,540.00 | 8,359 | 3,719 | 3,720.00 |
| | Placebo | M1.054 | NF54/7G8 | Yes/Yes | 462 | 41 | 1 | 1 | 1 | 1 | 1 | 32 | 1 | 1 |
| | | M1.065 | NF54/7G8 | Yes/No | 42 | *NS*[2] | *NS*[2] | *NS*[2] | 186 | 145 | 4 | 85 | 44 | 2 |
| | | M1.075 | NF54/7G8 | Yes/Yes | 35 | 1 | 1 | 1 | 1 | 1 | 1 | 187 | 152 | 5 |
| | | M1.058 | 7G8/NF54 | Yes/Yes | 53 | 62 | 12 | 1 | 67 | 1 | 1 | 359 | 13 | 1 |
| | | M1.067 | 7G8/NF54 | Yes/Yes | 61 | 74 | 9 | 1 | 60 | 14 | 1 | 75 | 307 | 7 |
| | | M1.073 | 7G8/NF54 | Yes/Yes | 54 | *NS*[2] | *NS*[2] | *NS*[2] | 29 | 1 | 1 | 331 | 277 | 6 |

| Group | PfSPZ/Dose | ID | CHMI | Infected | ISI | | | | | | | | | |
|---|---|---|---|---|---|---|---|---|---|---|---|---|---|---|
| | | | | | Reciprocal serum dilution for 80% inhibition | | | | | | | | | |
| | | | | | **Pre-Immune** | **2 weeks post 3rd dose** | **Net (Post-Pre)** | **Ratio (Post/Pre)** | **Pre-CHMI 1** | **Pre-CHMI 1 Net** | **Pre-CHMI 1 Ratio** | **Pre-CHMI 2** | **Pre-CHMI 2 Net** | **Pre-CHMI 2 Ratio** |
| **Verification** | **9.0 X $10^5$ PfSPZ in 1.0 mL administered DVI at Day 0, 7, and 28.** | M1.051 | NF54/7G8 | No/No | 9.19 | 101.85 | 92.66 | 11.08 | 60.77 | 51.57 | 6.61 | 53.63 | 44.44 | 5.83 |
| | | M1.060 | NF54/7G8 | Yes/No | 17.06 | 57.45 | 40.39 | 3.37 | 55.66 | 38.6 | 3.26 | 49.46 | 32.4 | 2.9 |
| | | M1.063 | NF54/7G8 | No/No | 6.25 | 70.57 | 64.32 | 11.29 | 60.93 | 54.68 | 9.75 | 167.16 | 160.91 | 26.75 |
| | | M1.070 | NF54/7G8 | No/No | 19.17 | 58.98 | 39.81 | 3.08 | 191.2 | 172.03 | 9.97 | 89.14 | 69.97 | 4.65 |
| | | M1.071 | NF54/7G8 | No/No | 17.29 | 94.68 | 77.39 | 5.48 | 77.13 | 59.83 | 4.46 | 115.56 | 98.27 | 6.68 |
| | | M1.056 | 7G8/NF54 | Yes/No | 16.81 | 57.74 | 40.93 | 3.44 | 86.71 | 69.91 | 5.16 | 44.88 | 28.08 | 2.67 |
| | | M1.049 | 7G8/NF54 | No/No | 13.33 | 111.94 | 98.61 | 8.4 | 79.27 | 65.95 | 5.95 | 64.65 | 51.33 | 4.85 |
| | | M1.050 | 7G8/NF54 | No/No | 23.08 | 238.64 | 215.56 | 10.34 | 67.25 | 44.18 | 2.91 | 117.89 | 94.81 | 5.11 |
| | | M1.064 | 7G8/NF54 | No/No | 56.65 | *NS*[2] | *NS*[2] | *NS*[2] | 218.79 | 162.14 | 3.86 | 179.77 | 123.12 | 3.17 |
| | | M1.086 | 7G8/NF54 | No/No | 1 | 58 | 57 | 58 | 108.76 | 107.76 | 108.76 | 261.1 | 260.1 | 261.1 |
| | | M1.076 | 7G8/NF54 | No/Yes | 14.05 | 79.13 | 65.08 | 5.63 | 77.01 | 62.96 | 5.48 | 41.06 | 27.01 | 2.92 |
| | | M1.059 | NF54/7G8 | Yes/Yes | 4.98 | 39.88 | 34.9 | 8.01 | 52.66 | 47.68 | 10.57 | 59.92 | 54.94 | 12.03 |
| | **Placebo** | M1.054 | NF54/7G8 | Yes/Yes | 9.02 | 1 | 1 | 0.11 | 1 | 1 | 0.11 | 1 | 1 | 0.11 |
| | | M1.065 | NF54/7G8 | Yes/No | 10.29 | *NS*[2] | *NS*[2] | *NS*[2] | 12.33 | 2.04 | 1.2 | 21.94 | 11.65 | 2.13 |
| | | M1.075 | NF54/7G8 | Yes/Yes | 9.52 | 11.53 | 2 | 1.21 | 7.32 | 1 | 0.77 | 16.13 | 6.61 | 1.69 |
| | | M1.058 | 7G8/NF54 | Yes/Yes | 9.67 | 1 | 1 | 0.1 | 9.47 | 1 | 0.98 | | 1 | 1 |
| | | M1.067 | 7G8/NF54 | Yes/Yes | 1 | 1 | 1 | 1 | 1 | 1 | 1 | 1 | 1 | 1 |
| | | M1.073 | 7G8/NF54 | Yes/Yes | 51.17 | *NS*[2] | *NS*[2] | *NS*[2] | 17.54 | 1 | 0.34 | 57.41 | 6.24 | 1.12 |

| Group | PfSPZ/Dose | ID | CHMI | Infected | ELISA | | | | | | |
|---|---|---|---|---|---|---|---|---|---|---|---|
| | | | | | PfCSP IgG OD 1.0 | | | | | | |
| | | | | | Pre-Immune | 1 day post 1st dose | 7 days post 2nd dose | 2 weeks post 3rd dose | 3 weeks post 3rd dose/Pre-CHMI1 | 7 weeks post 3rd dose | 9-10 weeks post 3rd dose/Pre-CHMI2 |
| Verification | 9.0 X $10^5$ PfSPZ in 1.0 mL administered DVI at Day 0, 7, and 28. | M1.051 | NF54/7G8 | No/No | 58 | 1 | 8,373 | 10,025 | 12,174 | 7,178 | 5,405 |
| | | M1.060 | NF54/7G8 | Yes/No | 11 | 34 | 82 | 8,526 | 4,618 | 4,367 | 4,661 |
| | | M1.063 | NF54/7G8 | No/No | 79 | 23 | 3,102 | 13,590 | 10,083 | 7,300 | 5,342 |
| | | M1.070 | NF54/7G8 | No/No | 49 | 19 | 50,152 | 48,674 | 57,880 | 35,364 | 11,274 |
| | | M1.071 | NF54/7G8 | No/No | 1 | 1 | 4,466 | 12,203 | 11,834 | 6,980 | 4,739 |
| | | M1.056 | 7G8/NF54 | Yes/No | 2 | 12 | 20,711 | 14,042 | 16,248 | 7,411 | 4,314 |
| | | M1.049 | 7G8/NF54 | No/No | 135 | 33 | 9,797 | 13,973 | 8,353 | 7,151 | 5,978 |
| | | M1.050 | 7G8/NF54 | No/No | 17 | 1 | 1,775 | 4,345 | 7,398 | 3,664 | 4,202 |
| | | M1.064 | 7G8/NF54 | No/No | 15 | 9 | 17,418 | *31,040* | 24,893 | 14,052 | 12,836 |
| | | M1.086 | 7G8/NF54 | No/No | 53 | 18 | 5,501 | 26,632 | 26,227 | 14,740 | 9,642 |
| | | M1.076 | 7G8/NF54 | No/Yes | 45 | 6 | 25,567 | 11,359 | 6,134 | 4,046 | 3,204 |
| | | M1.059 | NF54/7G8 | Yes/Yes | 49 | 17 | 1,547 | 10,475 | 6,025 | 5,444 | 4,418 |
