# Supplementary material for: A PfSPZ vaccine immunization regimen equally protective against homologous and heterologous controlled human malaria infection
Source: NPJ Vaccines. 2022 Aug 23;7:100. doi: 10.1038/s41541-022-00510-z (PMC9396563; doi:10.1038/s41541-022-00510-z)
Supplement: Supplementary file 2 — REPORTING SUMMARY [file 41541_2022_510_MOESM2_ESM.pdf]

nature portfolio

Corresponding author(s): Stephen L. Hoffman, Benjamin Mordmüller

Last updated by author(s): May 5, 2022

Reporting Summary

Nature Portfolio wishes to improve the reproducibility of the work that we publish. This form provides structure for consistency and transparency in reporting. For further information on Nature Portfolio policies, see our Editorial Policies and the Editorial Policy Checklist.

Statistics

For all statistical analyses, confirm that the following items are present in the figure legend, table legend, main text, or Methods section.

n/a	Confirmed	
☐	☒	The exact sample size (n) for each experimental group/condition, given as a discrete number and unit of measurement
☐	☒	A statement on whether measurements were taken from distinct samples or whether the same sample was measured repeatedly
☐	☒	The statistical test(s) used AND whether they are one- or two-sided<br>*Only common tests should be described solely by name; describe more complex techniques in the Methods section.*
☐	☒	A description of all covariates tested
☒	☐	A description of any assumptions or corrections, such as tests of normality and adjustment for multiple comparisons
☐	☒	A full description of the statistical parameters including central tendency (e.g. means) or other basic estimates (e.g. regression coefficient) AND variation (e.g. standard deviation) or associated estimates of uncertainty (e.g. confidence intervals)
☒	☐	For null hypothesis testing, the test statistic (e.g. F, t, r) with confidence intervals, effect sizes, degrees of freedom and P value noted<br>*Give P values as exact values whenever suitable.*
☐	☒	For Bayesian analysis, information on the choice of priors and Markov chain Monte Carlo settings
☒	☐	For hierarchical and complex designs, identification of the appropriate level for tests and full reporting of outcomes
☒	☐	Estimates of effect sizes (e.g. Cohen's d, Pearson's r), indicating how they were calculated

Our web collection on statistics for biologists contains articles on many of the points above.

Software and code

Policy information about availability of computer code

Data collection	No code was used
Data analysis	Data was analyzed using R, version 3.6.3

For manuscripts utilizing custom algorithms or software that are central to the research but not yet described in published literature, software must be made available to editors and reviewers. We strongly encourage code deposition in a community repository (e.g. GitHub). See the Nature Portfolio guidelines for submitting code & software for further information.

Data

Policy information about availability of data

All manuscripts must include a data availability statement. This statement should provide the following information, where applicable:

- Accession codes, unique identifiers, or web links for publicly available datasets
- A description of any restrictions on data availability
- For clinical datasets or third party data, please ensure that the statement adheres to our policy

The data supporting the findings of this study are available within the Article and its Supplementary Information and from the corresponding author upon reasonable request.

Field-specific reporting

Please select the one below that is the best fit for your research. If you are not sure, read the appropriate sections before making your selection.

☒ Life sciences ☐ Behavioural & social sciences ☐ Ecological, evolutionary & environmental sciences

For a reference copy of the document with all sections, see nature.com/documents/nr-reporting-summary-flat.pdf

Life sciences study design

All studies must disclose on these points even when the disclosure is negative.

Sample size	During optimization phase, decisions to move to the next regimen was based on the point estimate of efficacy, assuming 100% infectivity of 3,200 PfSPZ Challenge. This means if five or more out of six vaccine recipients (or four or more out of five, if there is a drop-out) were protected, efficacy is >75%.<br>The sample size for the verification phase was calculated using the function nBinomial of package gsDesign of R version 2.15.1, which estimates the sample size required to detect a difference between two rates. The objective of the verification phase was to assess safety and protective efficacy of the PfSPZ Vaccine compared to placebo. To be able to show, with a power of 80% and a two-tailed alpha of 5%, that 25% or less of immunized volunteers and 90% controls, allocated in a 2:1 ratio become infected, 12 immunized and 6 placebo-treated volunteers are required. An allocation ratio of 2:1 instead of 1:1 is chosen to maximize safety data on PfSPZ Vaccine and to minimize the number of research subjects acquiring malaria (all placebo-treated volunteers are expected to acquire malaria).
Data exclusions	No data was excluded
Replication	The study had two phases to reproduce (verify) the best regimen.
Randomization	Randomization was only done during verification phase. On the day of first immunization (I), immediately prior to injection, a member of the clinical team informed the formulator about the next volunteer's ID number. The formulator identified the volunteer on a randomization card and prepared the inoculum accordingly. Randomization cards for volunteers were sent by a third party, independent of investigator and the Sponsor, approximately 24 hours before administration. They informed the formulator about allocation to PfSPZ Vaccine and placebo (physiological saline) and about allocation to the sequence of P. falciparum strains used for CHMI. Both randomizations were computer-generated. A dedicated member of the formulation team, who was not involved in volunteer management or diagnostic activities, managed the randomization cards. A sealed copy of the randomization cards was given to the local safety monitor in case that unblinding is necessary.
Blinding	During the verification phase, the study was double-blinded (neither volunteer nor clinical and diagnostic teams are aware of intervention allocation). Only the PfSPZ formulation team was aware of allocation. No individual of the PfSPZ formulation team was involved in clinical or diagnostic activities. Syringes were labeled with the volunteer ID (VID), date and number of injection, but not with a label that indicates its content. Delivery of syringes from the PfSPZ formulation team to the clinical team was done without visual contact between formulator and clinical team. The prepared syringe with all necessary documentation was placed in front of the injection room and the clinical team was notified by ringing a bell or knocking on the door. PfSPZ Vaccine, PfSPZ Challenge and placebo are clear fluids and not distinguishable by appearance, odor or any other characteristic.

Reporting for specific materials, systems and methods

We require information from authors about some types of materials, experimental systems and methods used in many studies. Here, indicate whether each material, system or method listed is relevant to your study. If you are not sure if a list item applies to your research, read the appropriate section before selecting a response.

Materials & experimental systems

n/a	Involved in the study	
☒	☐	Antibodies
☒	☐	Eukaryotic cell lines
☒	☐	Palaeontology and archaeology
☒	☐	Animals and other organisms
☐	☒	Human research participants
☐	☒	Clinical data
☒	☐	Dual use research of concern

Methods

n/a	Involved in the study	
☒	☐	ChIP-seq
☒	☐	Flow cytometry
☒	☐	MRI-based neuroimaging

Human research participants

Policy information about studies involving human research participants

Population characteristics	Healthy adults 21 to 42 years old. 21 female, 15 male.
Recruitment	Volunteers were recruited by use of an advertisement form approved by the ethics committee and distributed or posted in

Recruitment	the following places:<br>• In public places with the agreement of the owner or proprietor<br>• Via interviews and presentations (e.g. presentations at lectures or invited seminars)<br>• Via university mailing list
Ethics oversight	Ethics Committee of the Medical Faculty and the University Clinics of the University of Tübingen

Note that full information on the approval of the study protocol must also be provided in the manuscript.

Clinical data

Policy information about clinical studies

All manuscripts should comply with the ICMJE guidelines for publication of clinical research and a completed CONSORT checklist must be included with all submissions.

Clinical trial registration	NCT02704533
Study protocol	Is supplement of the submission.
Data collection	Data was collected at the Institut für Tropenmedizin, Universitätsklinikum Tübingen. Enrollment took place from 22 September 2016 to 1 March 2018.
Outcomes	Primary safety endpoint<br>Number or occurrence of at least possibly related Grade 3 AEs and SAEs from time of first administration of PfSPZ Vaccine to the end of the follow-up period.<br><br>Primary safety endpoint PfSPZ Challenge (7G8) dose finding<br>Number or occurrence of at least possibly related Grade 3 AEs and SAEs from time of first administration of PfSPZ Challenge (7G8) to the end of the follow-up period.<br><br>Primary efficacy endpoint<br>Proportion of protected volunteers. Protection is defined as the absence of parasites in the peripheral blood (parasitemia) for 28 days following PfSPZ Challenge (NF54) or PfSPZ Challenge (7G8) injection. Parasitemia is defined as at least one qPCR result above 100 parasites per mL among three positive results at least 12 hours apart or as a positive thick blood smear.<br>During the optimization phase PfSPZ Challenge (NF54) will be used for CHMI. During verification phase PfSPZ Challenge (NF54) and PfSPZ Challenge (7G8) will be used consecutively in randomized sequence. In the verification phase, the proportion protected after the first of the two sequential CHMI's will be the primary endpoint.
